# Faunistic study of Coleoptera (Buprestidae, Carabidae, Cerambycidae, Lucanidae and Melyridae) on Gageodo Island, south-westernmost Korean Peninsula

**DOI:** 10.3897/BDJ.13.e146229

**Published:** 2025-03-31

**Authors:** Donguk Kim, Dooyoung Kim, Dongmin Kim, Young-Kun Kim, Sang Jae Suh, Kwang Shik Choi

**Affiliations:** 1 School of Life Sciences, BK21 FOUR KNU Creative BioResearch Group, Kyungpook National University, Daegu, Republic of Korea School of Life Sciences, BK21 FOUR KNU Creative BioResearch Group, Kyungpook National University Daegu Republic of Korea; 2 Department of Biology, College of Natural Sciences, Kyungpook National University, Daegu, Republic of Korea Department of Biology, College of Natural Sciences, Kyungpook National University Daegu Republic of Korea; 3 Department of Biomedical Convergence Science and Technology, School of Industrial Technology Advances, Kyungpook National University, Daegu, Republic of Korea Department of Biomedical Convergence Science and Technology, School of Industrial Technology Advances, Kyungpook National University Daegu Republic of Korea; 4 Department of Applied Biology, Kyungpook National University, Daegu, Republic of Korea Department of Applied Biology, Kyungpook National University Daegu Republic of Korea; 5 School of Applied Biosciences, Kyungpook National University, Daegu, Republic of Korea School of Applied Biosciences, Kyungpook National University Daegu Republic of Korea; 6 Department of Plant Medicine, College of Agricultural and Life Sciences, Kyungpook National University, Daegu, Republic of Korea Department of Plant Medicine, College of Agricultural and Life Sciences, Kyungpook National University Daegu Republic of Korea; 7 Institute of Plant Medicine, College of Agricultural and Life Sciences, Kyungpook National University, Daegu, Republic of Korea Institute of Plant Medicine, College of Agricultural and Life Sciences, Kyungpook National University Daegu Republic of Korea

**Keywords:** biodiversity, *Intybia*, island fauna, new record, Korean Archipelago, species inventory

## Abstract

**Background:**

The Korean Archipelago consists of more than 3,348 islands, many of which have an intact biodiversity. Gageodo Island, which is the south-westernmost island in the Peninsula, is characterised by floristic and faunistic features that are distinct from those of the mainland, making it of biogeographical and ecological interest. However, due to the difficulties associated with surveying this Island, it remains under-investigated. In particular, the Island's coleopteran fauna remains poorly understood.

**New information:**

In this study, the authors surveyed Buprestidae (jewel beetles), Carabidae (ground beetles), Cerambycidae (longhorn beetles), Lucanidae (stag beetles) and Melyridae (soft-winged flower beetles) on Gageodo Island. Each species was identified and ecological notes were recorded. To update the coleopteran list for the Island, previous studies that examined samples from Gageodo Island were compiled and organised. As a result, 31 species and three families were recorded on the Island for the first time, for a total of 93 species within 16 families. Of these, the melyrid species, *Intybiatsushimensis* (Satô & Ohbayashi, 1968) is reported for the first time in the Korean Peninsula. This study contributes to understand the coleopteran fauna of the biogeographically important Island in Korea and will serve as a foundational piece for understanding the fauna of Gageodo.

## Introduction

The Korean Peninsula is surrounded by more than 3,348 islands, many with a well-preserved biodiversity (Honam National Institute of Biological Resources (HNIBR) 2022). Studying island organisms is important for understanding colonisation, speciation and biodiversity conservation (Gillespie and Roderick 2002). In line with this, a recent revision of the insect fauna on the islands of Korea established a preliminary checklist for 541 islands with current insect records, with a total of 6,117 species recorded (Honam National Institute of Biological Resources (HNIBR) 2022).

**Figure 1.**
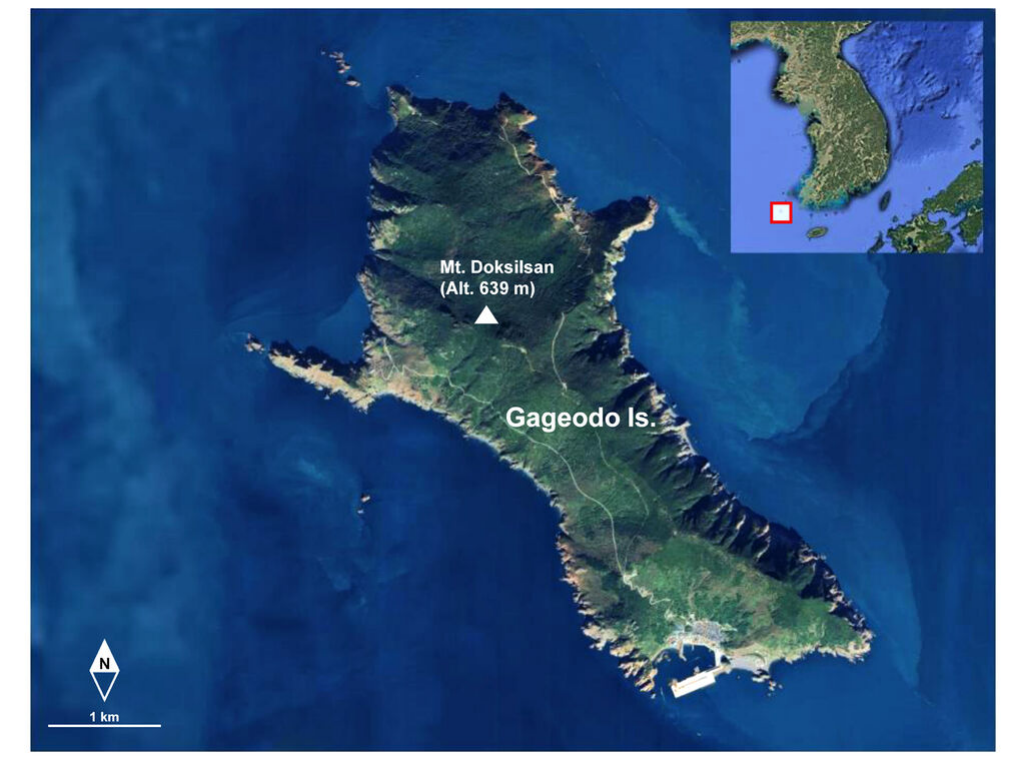
Location of Gageodo Island, South Korea. The map was taken from Google Earth (https://earth.google.com) and was edited using Adobe Photoshop 21.2.0 (Adobe Systems Inc.).

**Figure 2.**
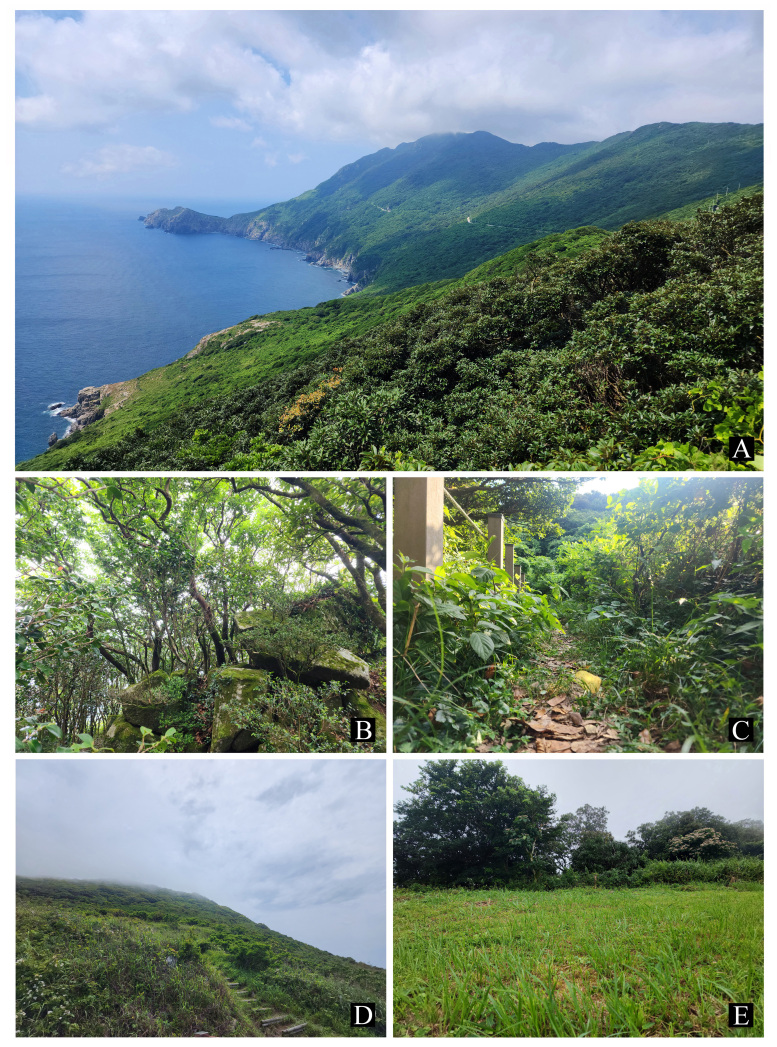
Gageodo Island. **A** View of Mt. Doksilsan; **B** Evergreen broad-leaved forest on Mt. Doksilsan; **C** Hiking trails; **D** Grassy hill; **E** Grasslands on Mt. Doksilsan.

Gageodo Island (also known as Soheuksando; 34°04'N, 125°06'E; area: 9.1 km²) is located approximately 120 km from the mainland and is the south-westernmost island in the Korean Peninsula (Fig. 1). The terrain of the Island is rugged, characterised by the steep slopes of Mt. Doksilsan (altitude: 639 m), which is located at the heart of the Island and is the third-highest non-mainland mountain in Korea (Fig. 2A). The summit of the mountain is adorned with temperate deciduous species such as maple and oak, while the lower areas are dominated by evergreen broad-leaved forest, resulting in diverse vegetation. The Island is strongly influenced by northwesterly seasonal winds in the spring and the summer North Pacific oceanic climate. The annual average of foggy days is approximately 280, with only around 70 clear days (Korea's average is 81 clear days), indicating a high frequency of fog in the region (Korea Forest Research Institute (KFRI) 2015, National Research Institute of Maritime Cultural Heritage of Korea (NRIMCH) 2018).

Even though the Korean Islands have been connected to the mainland during recent glacial periods, Gageodo Island harbours unique floristic and faunistic characteristics compared to the mainland, maybe due to its being isolated in the south-westernmost location in Korea (National Research Institute of Maritime Cultural Heritage of Korea (NRIMCH) 2018). For example, the following endemic species have been reported on the island: *Pseudocneorhinussoheuksandoensis* Han & Yoon, 2000 (Curculionidae), *Bryaxisnemorosus* Choi, Park, Lee & Park, 2023 (Staphylinidae), *Coleophorafasciella* Koo & Baldizzone, 2020 (Coleophoridae), *Gammarusgageoensis* Kim, Lee & Min, 2010 (Gammaridae), *Amynthasgageodo* Blakemore, 2012 (Megascolecidae), *Eiseniagaga* Blakemore & Park, 2012 (Lumbricidae), *Potentillagageodoensis* Kim, 2014 (Rosaceae), *Calantheinsularis* Oh, Suh & Park, 2015 (Orchidaceae) and *Calantherubra* Oh, Suh & Park, 2015 (Orchidaceae). Accordingly, Gageodo Island is of faunistic interest.

The first survey of the insect fauna on Gageodo Island was conducted by Shin and Noh (1970), who recorded seven orders, 34 families and 65 species, of which eight families and 24 species were from the order Coleoptera. Since this survey, only a few independent, isolated studies have been conducted (Table 1) and the list of coleopteran species on Gageodo Island remains unorganised and possibly incomplete because several coleopteran families that are common on the Korean Peninsula have not yet been documented on the Island.

In this study, we surveyed Buprestidae (jewel beetles), Carabidae (ground beetles), Cerambycidae (longhorn beetles), Lucanidae (stag beetles) and Melyridae (soft-winged flower beetles) on Gageodo Island. The results from previous faunistic and taxonomic studies on Gageodo Island were then combined with our identifications to provide an updated list of coleopteran species for the Island.

## Materials and methods

Three explorations were conducted on Gageodo Island in 2015 (July 27–28) and 2023 (May 26–28 and August 12–14). Specimens were collected using four methods: visual searches, sweeping, pit-fall traps and a light trap. Visual searches were conducted to collect specimens that were either resting or actively moving on flowers and leaves of plants, as well as those hiding under stones. Sweeping was conducted using an insect net (net diameter: 0.5 m; pole length: 3 m) to capture specimens by sweeping through herbaceous and woody vegetation. Pit-fall traps were set up by placing 40 plastic cups (diameter: 9.0 cm; height: 14.5 cm) at 10-metre intervals, each filled with molasses to attract beetles. A light trap was constructed using a rectangular frame (height: 1.5 m) covered with a white cloth. Inside the frame, a tripod (height: 1.0 m) was secured, supporting a 400 W mercury lamp. Specimens were collected that were attracted to the ultraviolet light.

Collected specimens were either dried and mounted or preserved in 95% ethanol (EtOH) for future study. They were then deposited in the Laboratory of Animal Systematics and Taxonomy, School of Life Sciences, College of Natural Sciences, Kyungpook National University (KNU, Daegu, Korea).

Morphological examinations were conducted using a stereoscopic microscope (Olympus SZX16, Tokyo, Japan). Photographs of the speciemens were taken using the Michrome 16 CMOS (Tucsen, Fujian, China) or the Olympus OMD EM10 Mark II digital camera.

The following catalogues are majorly referred for nomenclature: (1) Buprestidae (Löbl and Löbl 2016), (2) Carabidae (Löbl and Löbl 2017), (3) Cerambycidae (Danilevsky 2020), (4) Lucanidae (Löbl and Löbl 2016) and Melyridae (Löbl and Smetana 2007). The identification and nomenclature of each species were based on the following recent literature: (1) Buprestidae (Lee and Ahn 2012), (2) Carabidae (Habu 1967, Habu 1973, Habu 1978, Kwon and Lee 1984, Lafer and Kataev 2008, Park and Park 2013, Park et al. 2014, Fedorenko 2021, Li et al. 2024), (3) Cerambycidae (Yamasako and Ohbayashi 2012, Jang et al. 2015, Lee and Lee 2021), (4) Lucanidae (Kim and Kim 2014, Kim et al. 2019) and (5) Melyridae (Ikeda and Yoshitomi 2017).

## Checklists

### Checklist of coleopteran species on Gageodo Island

#### Coleoptera

C7E63421-AC4D-5FA5-A2EA-054FB321FF11

#### Anthribidae

B4550C7E-59AA-532C-8C0F-AC2659706744

#### Anthribinae

D51B8519-4A96-51D8-9B43-74BC55C7BDB6

#### Tropiderini

FE98AC54-799D-53F4-830D-A61C3FD4C473

#### Tropideres naevulus

Faust, 1887

96B017A8-2EE7-5158-9817-9472F3968324

##### Notes

Park et al. (2012) .

#### Bostrichidae

A66D8A90-E095-5683-A664-B0AFAC223B70

#### Polycaoninae

ABD6B1A3-E70B-547E-8443-A929D7F47FBC

#### Melalgus batillus

(Lesne, 1902)

118AADA8-5291-5F05-8B8C-7823FA63D1E2

##### Notes

Park et al. (2024) .

#### Brentidae

E6122598-339E-575C-9360-D34593097CBB

#### Apioninae

8F304522-5EA2-5F81-899B-40D0A79A2E4C

#### Apionini

DF0D76A0-7656-5CAD-90BB-ADD0A7C6F241

#### Sergiola (Sergiola) griseopubescens

(Roelofs, 1874)

49CD04E2-A4C1-54C2-BC87-9BD64D4E810C

##### Notes

Han et al. (2013) .

#### Buprestidae

205F558A-F92F-5365-B6E5-68289C3DB8E7

#### Agrilinae

D4561ED7-5F75-5666-A8E1-33932E2940E6

#### Agrilini

AF89FAC3-676B-5259-8410-0132AE849A93

#### Agrilus moerens

Saunders, 1873

B790B415-177E-5A2F-BCC2-90BF09F1D052

##### Materials

- **Type status:** Other material. **Occurrence:** sex: 1 male, 1 female; occurrenceID: 9EFBF9BB-F771-5D0F-AE57-51EFC2BD81BE; **Location:** island: Gageodo Island; country: South Korea; stateProvince: Jeollanam-do; locality: Shinan-gun, Heuksan-myeon, Gageodo-ri, 27.V.2023, Sang Jae Suh Coll.; **Identification:** identifiedBy: Donguk Kim; **Event:** eventDate: 27.V.2023; **Record Level:** basisOfRecord: Dried Specimens

##### Notes

This species was observed on a leaf of *Puerariamontana* (Fabaceae) growing on a sunny, grassy hill (Fig. 2D and Fig. 4). This is the first record of this species on Gageodo Island.

#### Carabidae

7661898D-6BE2-53ED-BF3C-07E6B75D27B1

#### Brachininae

2721F3EF-188C-568A-8EEF-50D6A6042CF6

#### Brachinini

2381A735-D3D4-5F96-B402-74F8F12B9A5C

#### Stenaptinus occipitalis jessoensis

(Morawitz, 1862)

EB3779A5-E338-5BD2-A919-D7D51C73461F

##### Materials

- **Type status:** Other material. **Occurrence:** sex: 1 female; occurrenceID: 89CDA6D2-76B0-5BF0-B47D-A3478EF000D9; **Location:** island: Gageodo Island; country: South Korea; stateProvince: Jeollanam-do; locality: Shinan-gun, Heuksan-myeon, Gageodo-ri, Sang Jae Suh Coll.; **Identification:** identifiedBy: Dooyoung Kim; **Event:** eventDate: 26.V.2023; **Record Level:** basisOfRecord: Dried Specimen

##### Notes

This species was collected during visual searches of the grasslands of low-lying coastal areas at night (Fig. 5A). This is the first record of this species on Gageodo Island.

#### Carabinae

7F8610B8-2425-52C2-BFB4-59DE4BB77DC1

#### Carabini

FAA06064-AB0E-56E6-964C-5C72E9B422C3

#### Calosoma (Calosoma) maximowiczi

(Morawitz, 1863)

5FD272F6-FA96-55E0-A77C-5E32F02AF8FC

##### Materials

- **Type status:** Other material. **Occurrence:** sex: 2 males, 2 females; occurrenceID: 7FC42F96-7760-552B-BE8C-D96251C637CC; **Location:** island: Gageodo Island; country: South Korea; stateProvince: Jeollanam-do; locality: Shinan-gun, Heuksan-myeon, Gageodo-ri, Donguk Kim leg.; **Identification:** identifiedBy: Donguk Kim; **Event:** eventDate: 28.VII.2015; **Record Level:** basisOfRecord: Dried Specimens
- **Type status:** Other material. **Occurrence:** sex: 1 male; occurrenceID: 7FC42F96-7760-552B-BE8C-D96251C637CC; **Location:** island: Gageodo Island; country: South Korea; stateProvince: Jeollanam-do; locality: Shinan-gun, Heuksan-myeon, Gageodo-ri, Sang Jae Suh Coll.; **Identification:** identifiedBy: Dooyoung Kim; **Event:** eventDate: 28.V.2023; **Record Level:** basisOfRecord: Dried Specimen
- **Type status:** Other material. **Occurrence:** sex: 1 male, 6 females; occurrenceID: 7FC42F96-7760-552B-BE8C-D96251C637CC; **Location:** island: Gageodo Island; country: South Korea; stateProvince: Jeollanam-do; locality: Shinan-gun, Heuksan-myeon, Gageodo-ri, Sang Jae Suh Coll.; **Identification:** identifiedBy: Dooyoung Kim; **Event:** eventDate: 13.VIII.2023; **Record Level:** basisOfRecord: Dried Specimens

##### Notes

This species was collected during visual searches and in pit-fall traps (Fig. 5B). Most of the individuals (n = 10) were observed on forest roads in the mid-altitude areas of Mt. Doksilsan (Fig. 2C). This is the first record of this species on Gageodo Island.

#### Harpalinae

339D3BA0-F5F0-5B1E-A753-338C6775E6F3

#### Chalaeniini

E729FEC7-AE15-5EFD-BEED-F11086DE1CAA

#### Chlaenius (Achlaenius) kurosawai

Kasahara, 1986

826460C3-62F5-54C7-B2CA-5EA570BFCB37

##### Materials

- **Type status:** Other material. **Occurrence:** sex: 2 males; occurrenceID: 28B2E255-801C-56AF-B836-39E31257729C; **Location:** island: Gagedo Island; country: South Korea; stateProvince: Jeollanam-do; locality: Shinan-gun, Heuksan-myeon, Gageodo-ri, Sang Jae Suh Coll.; **Identification:** identifiedBy: Dooyoung Kim; **Event:** eventDate: 26.V.2023; **Record Level:** basisOfRecord: Dried Specimens

##### Notes

This species was collected during visual searches of the grasslands of highland areas on Mt. Doksilsan (Fig. 2 and Fig. 5C). This is the first record of this species on Gageodo Island.

#### Chlaenius (Haplochlaenius) costiger costiger

Chaudoir, 1856

7C3E14FC-2109-5A8A-ACEB-BC57F8C07996

##### Materials

- **Type status:** Other material. **Occurrence:** sex: 1 male, 1 female; occurrenceID: 4EEE5155-0CB6-554E-A97D-C36B5128039D; **Location:** island: Gagedo Island; country: South Korea; stateProvince: Jeollanam-do; locality: Shinan-gun, Heuksan-myeon, Gageodo-ri, Sang Jae Suh Coll.; **Identification:** identifiedBy: Dooyoung Kim; **Event:** eventDate: 13.VIII.2023; **Record Level:** basisOfRecord: Dried Specimens

##### Notes

This species was collected during visual searches and was predominantly observed on forest roads in the mid-altitude areas of the Island (Fig. 5D). This is the first record of this species on Gageodo Island.

#### Chlaenius (Lissauchenius) naeviger naeviger

Morawitz, 1862

4CA2290A-DF2F-54D2-8592-E9FF0B36BA64

##### Materials

- **Type status:** Other material. **Occurrence:** sex: 12 males, 6 females; occurrenceID: 253A37A6-0045-5980-A85F-79DAD9E7DDBA; **Location:** island: Gagedo Island; country: South Korea; stateProvince: Jeollanam-do; locality: Shinan-gun, Heuksan-myeon, Gageodo-ri, Sang Jae Suh Coll.; **Identification:** identifiedBy: Dooyoung Kim; **Event:** eventDate: 26.V.2023; **Record Level:** basisOfRecord: Dried & Ethanol-Preserved Specimens

##### Notes

This species was collected during visual searches and in pit-fall traps. It was widely observed on forest roads from the lowlands to mid-mountainous areas of the Island (Fig. 2C and Fig. 5E). This is the first record of this species on Gageodo Island.

#### Chlaenius (Pachydinodes) virgulifer

Chaudoir, 1876

A3BD3A17-F75A-5A2E-BD65-E414FA779F75

##### Materials

- **Type status:** Other material. **Occurrence:** sex: 2 males; occurrenceID: D539A386-DC75-5C1E-996C-7687ADBF7800; **Location:** island: Gagedo Island; country: South Korea; stateProvince: Jeollanam-do; locality: Shinan-gun, Heuksan-myeon, Gageodo-ri, Sang Jae Suh Coll.; **Identification:** identifiedBy: Dooyoung Kim; **Event:** eventDate: 26.V.2023; **Record Level:** basisOfRecord: Dried Specimens

##### Notes

This species was collected during visual searches and was observed under the piles of leaves on forest roads in mid-altitude areas of the Island (Fig. 5F). This is the first record of this species on Gageodo Island.

#### Galeritini

ED4367B9-1E21-53BE-BDD8-1B386370D0FF

#### Galerita (Galerita) orientalis

Schmidt-Göbel, 1946

93277BFB-C545-50D3-B204-3877271BAF7D

##### Materials

- **Type status:** Other material. **Occurrence:** sex: 11 males, 10 females; occurrenceID: 36BE5CDC-D018-5A9D-984A-13D522A2E8EE; **Location:** island: Gagedo Island; country: South Korea; stateProvince: Jeollanam-do; locality: Shinan-gun, Heuksan-myeon, Gageodo-ri, Sang Jae Suh Coll.; **Identification:** identifiedBy: Dooyoung Kim; **Event:** eventDate: 27.V.2023; **Record Level:** basisOfRecord: Dried & Ethanol-Preserved Specimens

##### Notes

**Figure 3.**
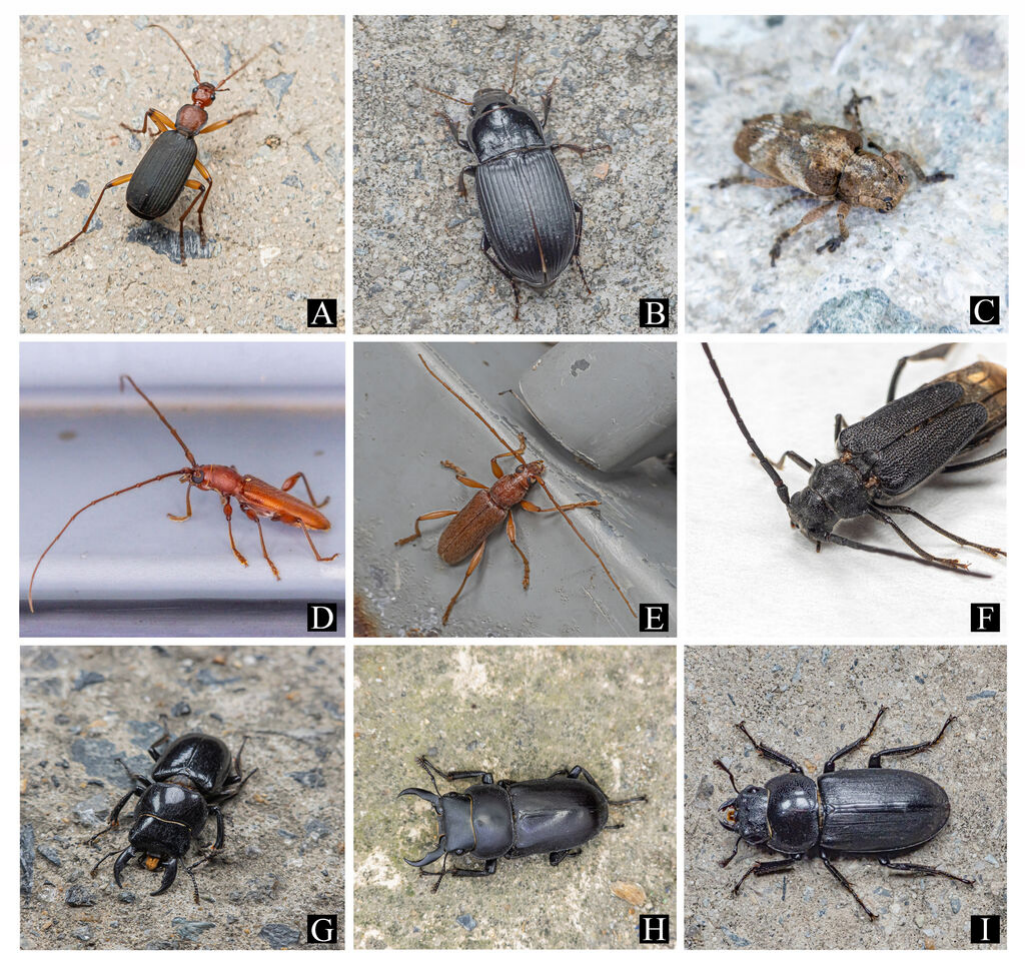
Beetles observed on Gageodo Island. **A** Galerita (Galerita) orientalis (Carabidae); **B** Harpalus (Haploharpalus) corporosus (Carabidae); **C** Pterolophia (Hylobrotus) annulata (Cerambycidae); **D** *Nysinarufescens* (Cerambycidae); **E** *Ceresiumflavipes* (Cerambycidae); **F** *Psephactusremigerremiger* (Cerambycidae); **G** *Dorcusconsentaneusconsentaneus* (Lucanidae); **H** *Dorcusrectusrectus* (Lucanidae); **I** *Dorcusrectusrectus* (Lucanidae).

**Figure 4.**
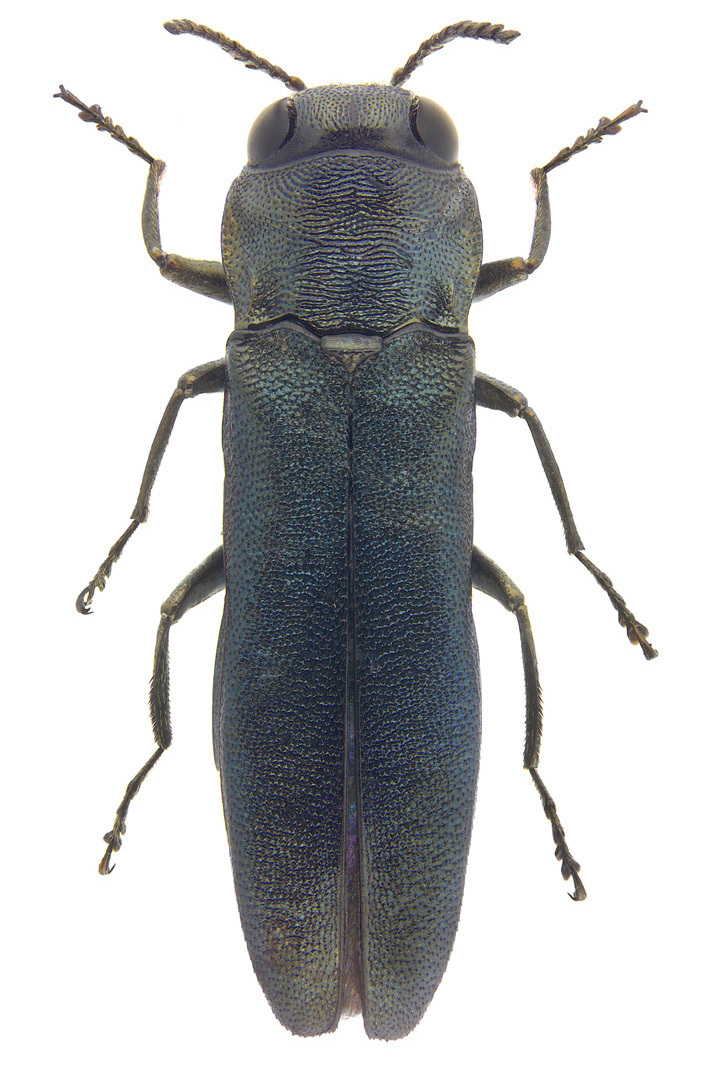
Buprestid species from Gageodo Island: *Agrilusmoerens*.

**Figure 5.**
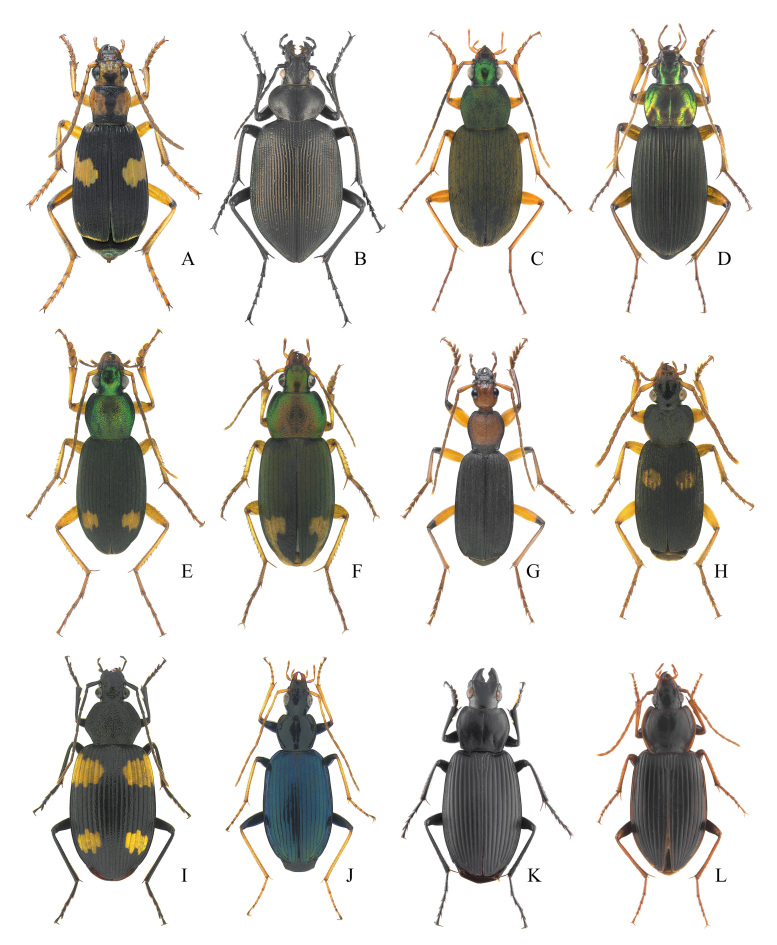
Carabid species from Gageodo Island. **A** *Stenaptinusoccipitalisjessoensis*; **B** Calosoma (Calosoma) maximowiczi; **C** Chlaenius (Achlaenius) kurosawai; **D** Chlaenius (Haplochlaenius) costiger costiger; **E** Chlaenius (Lissauchlaenius) naeviger naeviger; **F** Chlaenius (Pachydinodes) virgulifer; **G** Galerita (Galerita) orientalis; **H** Planetes (Planetes) puncticeps; **I** *Dischissusmirandus*; **J** *Dicranoncusfemoralis*; **K** Diplocheila (Submera) zeelandica; **L** Synuchus (Synuchus) cycloderus.

This species was collected during visual searches and in pit-fall traps. It was widely observed on forest roads in the lowlands of Gageodo Island (Fig. 3A and Fig. 5G). This is the first record of this species on Gageodo Island.

#### Planetes (Planetes) puncticeps

Andrewes, 1919

7F6AC113-33DF-5803-951E-6FF68146D0F7

##### Materials

- **Type status:** Other material. **Occurrence:** sex: 1 male; occurrenceID: 03E5BF39-FD61-538F-BFC6-AB965779B846; **Location:** island: Gagedo Island; country: South Korea; stateProvince: Jeollanam-do; locality: Shinan-gun, Heuksan-myeon, Gageodo-ri, Sang Jae Suh Coll.; **Identification:** identifiedBy: Dooyoung Kim; **Event:** eventDate: 26.V.2023; **Record Level:** basisOfRecord: Dried Specimen

##### Notes

This species was collected using pit-fall traps along hiking trails in mid-altitude areas of the Island (Fig. 2C and Fig. 5H). This is the first record of this species on Gageodo Island.

#### Harpalini

5C054F96-3249-5057-B45D-ABFE1E2E60CD

#### Anisodactylus (Pseudanisodactylus) punctatipennis

Morawitz, 1862

F9EF1EE8-82A2-5365-BA9B-EDF72F3C23CB

##### Materials

- **Type status:** Other material. **Occurrence:** sex: 2 males; occurrenceID: 314A2588-7D48-511C-9829-7991B87E2FCC; **Location:** island: Gagedo Island; country: South Korea; stateProvince: Jeollanam-do; locality: Shinan-gun, Heuksan-myeon, Gageodo-ri, Sang Jae Suh Coll.; **Identification:** identifiedBy: Dooyoung Kim; **Event:** eventDate: 14.VIII.2023; **Record Level:** basisOfRecord: Dried Specimens

##### Notes

**Figure 6.**
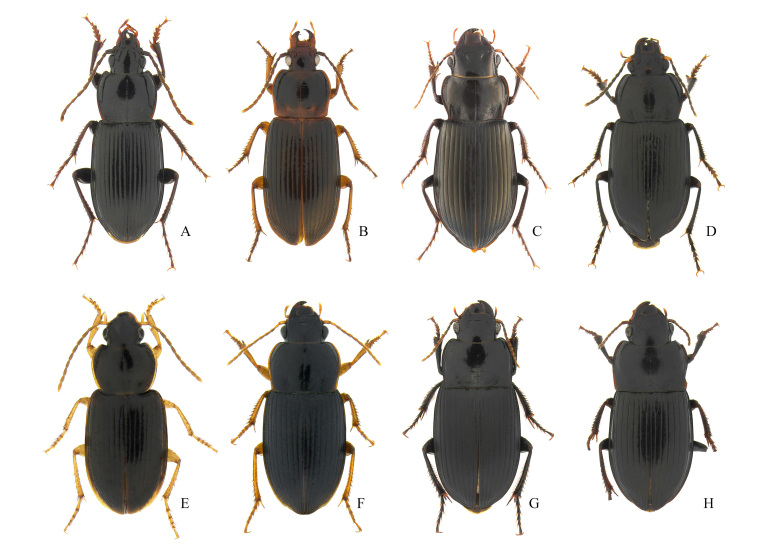
Carabid species from Gageodo Island. **A** Pterostichus (Rhagadus) solskyi; **B** *Oxycentrusargutoroides*; **C** Amara (Curtonotus) giganteus; **D** Anisodactylus (Pseudanisodactylus) punctatipennis; **E** Stenolophus (Astenolophus) fulvicornis; **F** Harpalus (Zangoharpalus) tinctulus; **G** Harpalus (Haploharpalus) corporosus; **H** *Nipponoharpalusdiscrepans*.

This species was collected using pit-fall traps along the hiking trails of mid-altitude areas of the Island (Fig. 2C and Fig. 6D). This is the first record of this species on Gageodo Island.

#### Harpalus (Haploharpalus) corporosus

(Motschulsky, 1861)

27BA1AB3-98B5-5596-84A2-0F5965E3A543

##### Materials

- **Type status:** Other material. **Occurrence:** sex: 1 female; occurrenceID: 4F748C3D-DE5F-5A71-945E-8FE41389CD3E; **Location:** island: Gagedo Island; country: South Korea; stateProvince: Jeollanam-do; locality: Shinan-gun, Heuksan-myeon, Gageodo-ri, Sang Jae Suh Coll.; **Identification:** identifiedBy: Dooyoung Kim; **Event:** eventDate: 13.VIII.2023; **Record Level:** basisOfRecord: Dried Specimen

##### Notes

This species was collected during visual searches and was observed in the grasslands of low-lying coastal areas at night (Fig. 3B and Fig. 6G). This is the first record of this species on Gageodo Island.

#### Harpalus (Zangoharpalus) tinctulus

Bates, 1873

0A215C1B-0895-5846-8D20-0D86B74B92BD

##### Materials

- **Type status:** Other material. **Occurrence:** sex: 1 female; occurrenceID: 96329410-04B0-59B6-863E-E7DB7024F56E; **Location:** island: Gagedo Island; country: South Korea; stateProvince: Jeollanam-do; locality: Shinan-gun, Heuksan-myeon, Gageodo-ri, Sang Jae Suh Coll.; **Identification:** identifiedBy: Dooyoung Kim; **Event:** eventDate: 27.V.2023; **Record Level:** basisOfRecord: Dried Specimen

##### Notes

This species was collected using pit-fall traps along the hiking trails of mid-altitude areas of the Island (Fig. 2C and Fig. 6F). This is the first record of this species on Gageodo Island.

#### Nipponoharpalus discrepans

(Morawitz, 1862)

FF8FC8BD-A81D-548E-92C9-09C9BA674FFD

##### Materials

- **Type status:** Other material. **Occurrence:** sex: 1 female; occurrenceID: 633A2B5E-E628-5F0B-A397-BEB8C14891F4; **Location:** island: Gagedo Island; country: South Korea; stateProvince: Jeollanam-do; locality: Shinan-gun, Heuksan-myeon, Gageodo-ri, Sang Jae Suh Coll.; **Identification:** identifiedBy: Dooyoung Kim; **Event:** eventDate: 28.V.2023; **Record Level:** basisOfRecord: Dried Specimen

##### Notes

This species was collected using pit-fall traps along the hiking trails of mid-altitude areas of the Island (Fig. 2C and Fig. 6H). This is the first record of this species on Gageodo Island.

#### Oxycentrus (Oxycentrus) argutoroides

(Bates, 1873)

ED561090-1BFF-54E6-B6CE-0CF89B0E5FE3

##### Materials

- **Type status:** Other material. **Occurrence:** sex: 1 female; occurrenceID: FE02EDB2-29A4-5DB6-BA5D-A1F741CEDB29; **Location:** island: Gagedo Island; country: South Korea; stateProvince: Jeollanam-do; locality: Shinan-gun, Heuksan-myeon, Gageodo-ri, Sang Jae Suh Coll.; **Identification:** identifiedBy: Dooyoung Kim; **Event:** eventDate: 14.VIII.2023; **Record Level:** basisOfRecord: Dried Specimen

##### Notes

This species was collected under rocks in the grasslands of highland areas of Mt. Doksilsan (Fig. 2E and Fig. 6B). This species has both brachypterous and macropterous forms (Habu 1973) and the specimen examined here was brachypterous. This is the first record of this species on Gageodo Island.

#### Stenolophus (Astenolophus) fulvicornis

Bates, 1873

FA3B952E-6123-585C-A0B0-EB2EE54A3D89

##### Materials

- **Type status:** Other material. **Occurrence:** sex: 1 male; occurrenceID: D868CC44-35FA-59CA-955F-502C63A6BEEB; **Location:** island: Gagedo Island; country: South Korea; stateProvince: Jeollanam-do; locality: Shinan-gun, Heuksan-myeon, Gageodo-ri, Sang Jae Suh Coll.; **Identification:** identifiedBy: Dooyoung Kim; **Event:** eventDate: 27.V.2023; **Record Level:** basisOfRecord: Dried Specimen

##### Notes

This species was collected using pit-fall traps along the hiking trails in mid-altitude areas of the Island (Fig. 2C and Fig. 6E). This is the first record of this species on Gageodo Island.

#### Licinini

41092875-7B87-5238-AF73-BCFF9F1EB856

#### Diplocheila (Submera) zeelandica

(Redtenbacher, 1868)

3F71013C-6EA8-592A-B08E-D21585B2655D

##### Materials

- **Type status:** Other material. **Occurrence:** sex: 1 male; occurrenceID: 30D471A8-EFE3-5A0A-8AAC-6ECB61EB86D8; **Location:** island: Gagedo Island; country: South Korea; stateProvince: Jeollanam-do; locality: Shinan-gun, Heuksan-myeon, Gageodo-ri, Sang Jae Suh Coll.; **Identification:** identifiedBy: Dooyoung Kim; **Event:** eventDate: 14.VIII.2023; **Record Level:** basisOfRecord: Dried Specimen

##### Notes

A dead specimen of this species was collected along the hiking trails of mid-altitude areas of the Island (Fig. 5K). This is the first record of this species on Gageodo Island.

#### Panagaeini

A34C126E-18FD-5E1F-939B-4AB8B607821B

#### Dischissus mirandus

Bates, 1873

F0FF5D7E-26A6-5FEF-8AB9-02100F15835B

##### Materials

- **Type status:** Other material. **Occurrence:** sex: 3 females; occurrenceID: A3258E20-424E-5454-B485-C27E6C34CCD7; **Location:** island: Gagedo Island; country: South Korea; stateProvince: Jeollanam-do; locality: Shinan-gun, Heuksan-myeon, Gageodo-ri, Sang Jae Suh Coll.; **Identification:** identifiedBy: Dooyoung Kim; **Event:** eventDate: 28.V.2023; **Record Level:** basisOfRecord: Dried & Ethanol-Preserved Specimens

##### Notes

This species was collected during visual searches. It was observed on forest roads or hiking trails from the lowlands to the highland areas of Gageodo Island (Fig. 2B and Fig. 5I). This is the first record of this species on Gageodo Island.

#### Platynini

5E83B6E2-033B-5DEE-A374-ED3332E83AF2

#### Dicranoncus femoralis

Chaudoir, 1850

4A366E70-222C-5BB5-9B6B-40BE312B7F47

##### Materials

- **Type status:** Other material. **Occurrence:** sex: 10 males, 5 females; occurrenceID: AFA6A977-FF23-5C5D-B426-62F067D6BE20; **Location:** island: Gagedo Island; country: South Korea; stateProvince: Jeollanam-do; locality: Shinan-gun, Heuksan-myeon, Gageodo-ri, Sang Jae Suh Coll.; **Identification:** identifiedBy: Dooyoung Kim; **Event:** eventDate: 13.VIII.2023; **Record Level:** basisOfRecord: Dried & Ethanol-Preserved Specimens

##### Notes

This species was collected by sweeping and beating various broad-leaved trees that grow on forest roads or hiking trails, from the mid-mountainous areas to the highland areas of Mt. Doksilsan (Fig. 2B and Fig. 5J). This is the first record of this species on Gageodo Island.

#### Pterostichini

69B64921-7C01-5A0D-8571-04FFE56B0B57

#### Pterostichus (Rhagadus) solskyi

(Chaudoir, 1878)

2709E4D4-7E0D-5AE2-A32F-5CC281E71726

##### Materials

- **Type status:** Other material. **Occurrence:** sex: 4 males, 9 females; occurrenceID: F40F5F1E-A3AC-5F33-8A94-0404D7B45F80; **Location:** island: Gagedo Island; country: South Korea; stateProvince: Jeollanam-do; locality: Shinan-gun, Heuksan-myeon, Gageodo-ri, Sang Jae Suh Coll.; **Identification:** identifiedBy: Dooyoung Kim; **Event:** eventDate: 13.VIII.2023; **Record Level:** basisOfRecord: Dried & Ethanol-Preserved Specimens

##### Notes

This species was collected using pit-fall traps along the hiking trails in mid-altitude areas of the Island. It is a brachypterous species and represents the first record of this species on Gageodo Island (Fig. 6A).

#### Sphodrini

9372720B-F875-5D35-B714-AF5CD1A5E4A7

#### Synuchus (Synuchus) cycloderus

(Bates, 1873)

4F6CBDA1-3269-5B43-A1BA-073B5F93AD70

##### Materials

- **Type status:** Other material. **Occurrence:** sex: 1 female; occurrenceID: 0272A8B6-1970-5653-8FF5-AAE88410FC01; **Location:** island: Gagedo Island; country: South Korea; stateProvince: Jeollanam-do; locality: Shinan-gun, Heuksan-myeon, Gageodo-ri, Sang Jae Suh Coll.; **Identification:** identifiedBy: Dooyoung Kim; **Event:** eventDate: 28.V.2023; **Record Level:** basisOfRecord: Dried Specimen

##### Notes

This species was collected using pit-fall traps along the hiking trails in mid-altitude areas of the Island (Fig. 2C and Fig. 5L). This is the first record of this species on Gageodo Island.

#### Zabrini

CF1952B3-7C4E-5767-B5FB-837625E4EA2B

#### Amara (Curtonotus) giganteus

(Motschulsky, 1844)

120069C1-18DE-5385-981F-10271BA3F5DE

##### Materials

- **Type status:** Other material. **Occurrence:** sex: 1 female; occurrenceID: 825F17A7-216B-53D3-A2C7-B38A424C52B0; **Location:** island: Gagedo Island; country: South Korea; stateProvince: Jeollanam-do; locality: Shinan-gun, Heuksan-myeon, Gageodo-ri, Sang Jae Suh Coll.; **Identification:** identifiedBy: Dooyoung Kim; **Event:** eventDate: 28.V.2023; **Record Level:** basisOfRecord: Dried Specimen

##### Notes

This species was collected during visual searches and observed in the grasslands of low-lying coastal areas at night. This is the first record of this species on Gageodo Island (Fig. 6C).

#### Cerambycidae

120D9A4C-24FA-5485-8723-45DE5C9C2C3C

#### Cerambycinae

26386C19-516B-5D84-83BA-C35EE0C8C3D6

#### Callidiopini

F76349A8-DF09-564C-A5BE-A900A1EF9445

#### Ceresium flavipes

Fabricius, 1792

E5FE4E67-3344-59AA-8238-736F24155CD6

##### Materials

- **Type status:** Other material. **Occurrence:** sex: 2 males, 2 females; occurrenceID: D0F2839E-5E6E-5B68-B9F0-FBD8ED03F410; **Location:** island: Gagedo Island; country: South Korea; stateProvince: Jeollanam-do; locality: Shinan-gun, Heuksan-myeon, Gageodo-ri, Sang Jae Suh Coll.; **Identification:** identifiedBy: Donguk Kim; **Event:** eventDate: 13.VIII.2023; **Record Level:** basisOfRecord: Dried Specimens

##### Notes

**Figure 7.**
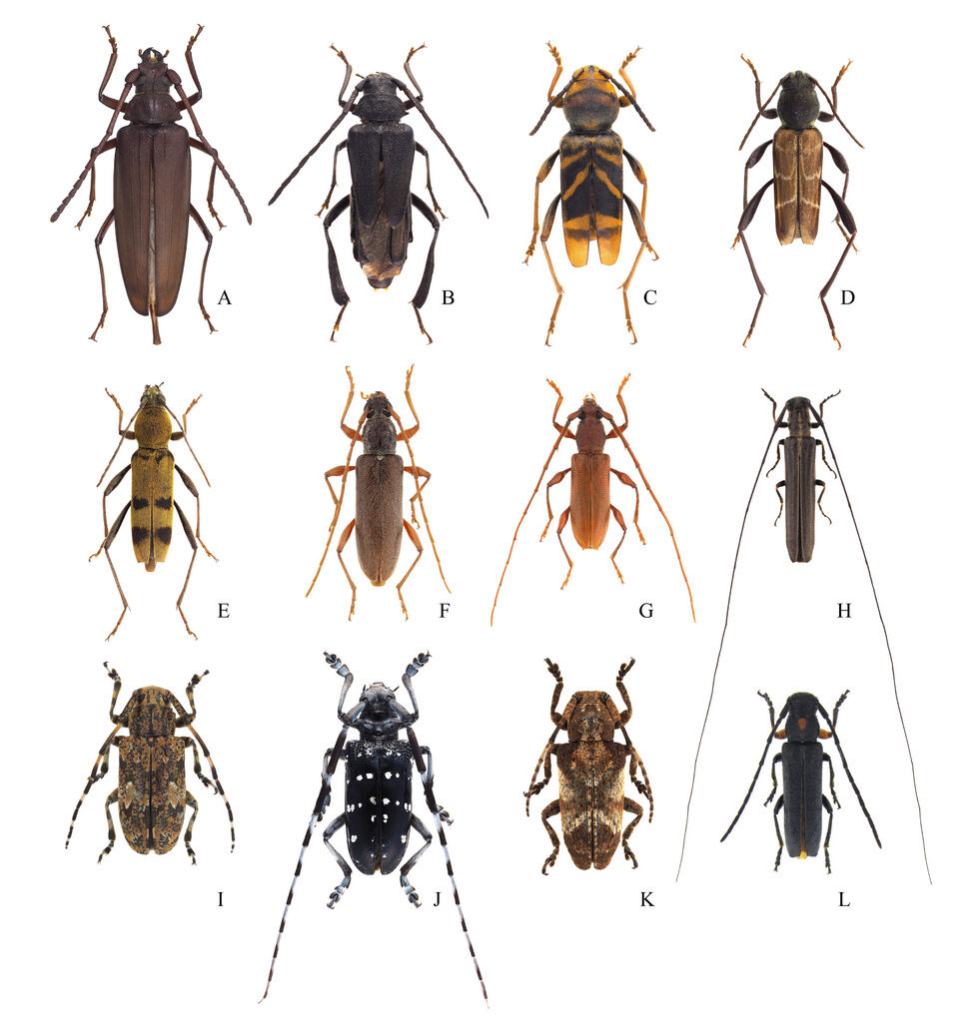
Cerambycid species from Gageodo Island. **A** *Aegosomasinicumsinicum*; **B** *Psephactusremigerremiger*; **C** Xylotrechus (Xyloclytus) chinensis; **D** Xylotrechus (Xylotrechus) cuneipennis; **E** *Chlorophorusmuscosus*; **F** *Ceresiumflavipes*; **G** *Nysinarufescens*; **H** *Pseudocalamobiusjaponicus*; **I** Agelasta (Dissosira) perplexa; **J** *Anoplophorachinensis*; **K** Pterolophia (Hylobrotus) annulata; **L** Phytoecia (Phytoecia) rufiventris.

This species was collected during visual searches and some individuals were attracted to street lights in low-lying coastal areas (Fig. 3E and Fig. 7F). This is the first record of this species on Gageodo Island.

#### Clytini

A510F7CF-7238-5239-A157-B9A2D6020ECD

#### Chlorophorus muscosus

(Bates, 1873)

E09C429E-8F84-591D-8C77-C0A9DCF31904

##### Materials

- **Type status:** Other material. **Occurrence:** sex: 1 female; occurrenceID: FF944C06-AD34-59D8-8A33-419837A87DA3; **Location:** island: Gagedo Island; country: South Korea; stateProvince: Jeollanam-do; locality: Shinan-gun, Heuksan-myeon, Gageodo-ri, Donguk Kim leg.; **Identification:** identifiedBy: Donguk Kim; **Event:** eventDate: 28.VII.2015; **Record Level:** basisOfRecord: Dried Specimen
- **Type status:** Other material. **Occurrence:** sex: 1 male; occurrenceID: FF944C06-AD34-59D8-8A33-419837A87DA3; **Location:** island: Gagedo Island; country: South Korea; stateProvince: Jeollanam-do; locality: Shinan-gun, Heuksan-myeon, Gageodo-ri, Sang Jae Suh Coll.; **Identification:** identifiedBy: Donguk Kim; **Event:** eventDate: 14.VIII.2023; **Record Level:** basisOfRecord: Dried Specimen

##### Notes

This species was collected through visual searching on the leaves of *Mallotusjaponicus* (Euphorbiaceae) growing along forest roads in the mid-altitude areas of Mt. Doksilsan (Fig. 7E). It was recorded in Gageodo Island by Lee (1987) and Hwang (2015).

#### Perissus kimi

Niisato & Koh, 2003

0233B15F-0476-50D4-A25F-099BB01C1D7D

##### Notes

Jang et al. (2015) .

#### Xylotrechus (Xyloclytus) chinensis

(Chevrolat, 1852)

F4924ABB-C520-525D-8F89-3943C54BBE5E

##### Materials

- **Type status:** Other material. **Occurrence:** sex: 1 female; occurrenceID: 2D87FC65-9B21-57CB-A62E-1614C506DCE8; **Location:** island: Gagedo Island; country: South Korea; stateProvince: Jeollanam-do; locality: Shinan-gun, Heuksan-myeon, Gageodo-ri, Sang Jae Suh Coll.; **Identification:** identifiedBy: Donguk Kim; **Event:** eventDate: 14.VIII.2023; **Record Level:** basisOfRecord: Dried Specimen

##### Notes

A dead specimen of this species was collected in mid-altitude areas of Mt. Doksilsan. This is the first record of this species on Gageodo Island (Fig. 7C).

#### Xylotrechus (Xylotrechus) cuneipennis

(Kraatz, 1879)

1642E46D-63A6-51BC-9D44-54C93DC04D88

##### Materials

- **Type status:** Other material. **Occurrence:** sex: 1 male, 1 female; occurrenceID: AFAF9D7C-249D-5F34-ABF6-D129F22E5D51; **Location:** island: Gagedo Island; country: South Korea; stateProvince: Jeollanam-do; locality: Shinan-gun, Heuksan-myeon, Gageodo-ri, Donguk Kim leg.; **Identification:** identifiedBy: Donguk Kim; **Event:** eventDate: 28.VII.2015; **Record Level:** basisOfRecord: Dried Specimens

##### Notes

This species was collected during visual searches. It was mating on a strut on *Machilusthunbergii* (Lauraceae), which was growing on a mountain ridge. This is the first record of this species on Gageodo Island (Fig. 7D).

#### Phoracanthini

65795952-5B92-56F4-A8A7-8090C1EA08F6

#### Nysina rufescens

(Pic, 1923)

9E713F27-7269-5812-B872-21934809B9C1

##### Materials

- **Type status:** Other material. **Occurrence:** sex: 1 male, 2 females; occurrenceID: 79442221-4AA1-5596-A6C6-6BDDF39B9F0D; **Location:** island: Gagedo Island; country: South Korea; stateProvince: Jeollanam-do; locality: Shinan-gun, Heuksan-myeon, Gageodo-ri, Sang Jae Suh Coll.; **Identification:** identifiedBy: Donguk Kim; **Event:** eventDate: 27.V.2023; **Record Level:** basisOfRecord: Dried & Ethanol-Preserved Specimens

##### Notes

This species was collected during visual searches. It was observed being active on *Machilusthunbergii* (Lauraceae) branches growing on low-lying coastal areas at night. Additionally, some individuals (n = 3) were attracted to street lights (Fig. 3D and Fig. 7G). It was recorded in Gageodo Island by Jang et al. (2015) and Lee and Lee (2021).

#### Lamiinae

213224EB-A522-51A4-B1E8-4A3B352DBF20

#### Acanthocinini

DD043375-4084-50AD-BDCF-BE08C919B9DA

#### Rondibilis (Rondibilis) undulata

(Pic, 1922)

BCA22CC8-9B57-5DE7-86C0-D4E297B375FA

##### Notes

Jang et al. (2015) .

#### Agapanthiini

4F08B7AC-A044-54F7-B800-E40854528603

#### Agapanthia (Amurobia) amurensis

Kraatz, 1879

A9C6E0C9-C5FB-5890-9CC0-53AD44244B16

##### Notes

Honam National Institute of Biological Resources (HNIBR) (2022) .

#### Pseudocalamobius japonicus

(Bates, 1873)

09452D85-2D6F-5BAC-AE95-092A6FCF5F8B

##### Materials

- **Type status:** Other material. **Occurrence:** sex: 1 female; occurrenceID: 1C1FC217-A8FC-5DC5-841A-447F6EBA15BA; **Location:** island: Gagedo Island; country: South Korea; stateProvince: Jeollanam-do; locality: Shinan-gun, Heuksan-myeon, Gageodo-ri, Sang Jae Suh Coll.; **Identification:** identifiedBy: Donguk Kim; **Event:** eventDate: 27.V.2023; **Record Level:** basisOfRecord: Dried Specimen

##### Notes

This species was collected by sweeping along the hiking trails in low-lying areas (Fig. 7H). This is the first record of this species on Gageodo Island.

#### Apomecynini

AA97449F-8E0C-56B6-A3DB-87C9E3C6CEB7

#### Ropica coreana

Breuning, 1980

9CC0544F-F2B7-5FCC-832A-8EAC0A96FFE8

##### Notes

Lee (1987), Hwang (2015).

#### Ceroplesini

9B285C6F-044C-5635-86B6-B15A4CE99D24

#### Moechotypa diphysis

(Pascoe, 1871)

26C4F99F-CB9B-5473-A84B-5C01C7016EEE

##### Notes

Honam National Institute of Biological Resources (HNIBR) (2022) .

#### Exocentrus (Exocentrus) lineatus

Bates, 1873

F56F8CE6-EEAF-552E-BB00-AE6C992AA9F0

##### Notes

Hwang (2015) .

#### Mesosini

C033E543-2C3B-5249-A0FA-86085AAFD07B

#### Agelasta (Dissosira) perplexa

(Pascoe, 1858)

25391522-7E45-55FE-9C8A-0C944C9ACC6C

##### Materials

- **Type status:** Other material. **Occurrence:** sex: 2 females; occurrenceID: 210127CC-92E7-59F8-A35E-2628184EE9C7; **Location:** island: Gagedo Island; country: South Korea; stateProvince: Jeollanam-do; locality: Shinan-gun, Heuksan-myeon, Gageodo-ri, Sang Jae Suh Coll.; **Identification:** identifiedBy: Donguk Kim; **Event:** eventDate: 14.VIII.2023; **Record Level:** basisOfRecord: Dried Specimens

##### Notes

This species was attracted to street lights in low-lying coastal areas and, in terms of its Korean distribution, it has only been recorded on Gageodo Island to date (Fig. 7I). Previously, this species was known to be active from late May to mid-July (Jang et al. 2015), but this survey confirmed that it remained active until mid-August. It was recorded in Gageodo Island by Kang (2002), Jang et al. (2015) and Hwang (2015).

#### Monochamini

058E3925-ABE9-546B-BD02-9BC0D579311D

#### Anoplophora chinensis

(Förster, 1771)

5BE9F5A2-A8F4-5E27-A87E-2C0D0B7AC47A

##### Materials

- **Type status:** Other material. **Occurrence:** sex: 1 male; occurrenceID: 3CD1570A-9E8D-5C68-9C20-D663B76AF31A; **Location:** island: Gageodo Island; country: South Korea; stateProvince: Jeollanam-do; locality: Shinan-gun, Heuksan-myeon, Gageodo-ri, Sang Jae Suh Coll.; **Identification:** identifiedBy: Donguk Kim; **Event:** eventDate: 14.VIII.2023; **Record Level:** basisOfRecord: Dried Specimen

##### Notes

This species was attracted to street lights in low-lying coastal areas (Fig. 7J). This is the first record of this species on Gageodo Island.

#### Phytoeciini

1EF20CEE-6D1A-5642-B5A8-9E4261F5965B

#### Phytoecia (Phytoecia) rufiventris

Gautier des Gottes, 1870

E633C0BE-A7D7-59F6-BF9A-EC2A44BB97EB

##### Materials

- **Type status:** Other material. **Occurrence:** sex: 2 males, 1 female; occurrenceID: 6E4CC22D-6B73-591E-AE70-C12BF9CDBAF4; **Location:** island: Gageodo Island; country: South Korea; stateProvince: Jeollanam-do; locality: Shinan-gun, Heuksan-myeon, Gageodo-ri, Sang Jae Suh Coll.; **Identification:** identifiedBy: Donguk Kim; **Event:** eventDate: 26.V.2023; **Record Level:** basisOfRecord: Dried Specimen

##### Notes

This species was collected during a visual search of *Artemisiaprinceps* (Asteraceae), growing on a coastal cliff (Fig. 7L). This is the first record of this species on Gageodo Island.

#### Pteropliini

E5DCE03C-3AEB-59B8-AA35-F516BEC7DF0D

#### Pterolophia (Hylobrotus) annulata

(Chevrolat, 1845)

5D3238F6-DB88-5B2D-9136-9D19E8588D2B

##### Materials

- **Type status:** Other material. **Occurrence:** sex: 2 females; occurrenceID: C1212CB2-7774-5570-AA04-04B33A24093E; **Location:** island: Gageodo Island; country: South Korea; stateProvince: Jeollanam-do; locality: Shinan-gun, Heuksan-myeon, Gageodo-ri, Sang Jae Suh Coll.; **Identification:** identifiedBy: Donguk Kim; **Event:** eventDate: 27.V.2023; **Record Level:** basisOfRecord: Dried Specimens

##### Notes

This species was attracted to street lights in low-lying coastal areas (Fig. 3C and Fig. 7K). It was first recorded in Gageodo Island by Hwang (2015).

#### Pterolophia (Pterolophia) angusta multinotata

(Pic, 1931)

B3A5E38A-F3AB-5110-87E6-F99523225345

##### Notes

Lee (1987) .

#### Lepturinae

A69F3962-A831-5E58-9EB8-19E2C6931225

#### Lepturini

C4FF01DD-7B33-550C-B5BA-0A06F9754709

#### Leptura (Leptura) annularis

Fabricius, 1801

AC059508-89C6-5BD3-9377-88F702B28CBF

##### Notes

Hwang (2015) .

#### Prioninae

7E495BCB-F423-5887-AF2C-1C6E4AA42882

#### Aegosomatini

F9C3F7A1-4747-5C11-AE63-3C81DFC7C452

#### Aegosoma sinicum sinicum

White, 1853

9F2AE554-3F35-5AD4-AA45-EDEFD2BA8088

##### Materials

- **Type status:** Other material. **Occurrence:** sex: 1 female; occurrenceID: 50D12E91-D524-54FF-B42C-D50C7ABB3F79; **Location:** island: Gageodo Island; country: South Korea; stateProvince: Jeollanam-do; locality: Shinan-gun, Heuksan-myeon, Gageodo-ri, Donguk Kim leg.; **Identification:** identifiedBy: Donguk Kim; **Event:** eventDate: 28.VII.2015; **Record Level:** basisOfRecord: Dried Specimen

##### Notes

This species was attracted to light traps in the forests of low-lying coastal areas (Fig. 7A). This is the first record of this species on Gageodo Island.

#### Anacolini

B93ECE52-C1F5-5560-853E-AC3E920B8E62

#### Psephactus remiger remiger

Harold, 1879

2A911443-7246-5114-908E-2CD3B36A3439

##### Materials

- **Type status:** Other material. **Occurrence:** sex: 2 females; occurrenceID: CA6E8ED0-135C-5137-AE9C-1C3788731958; **Location:** island: Gageodo Island; country: South Korea; stateProvince: Jeollanam-do; locality: Shinan-gun, Heuksan-myeon, Gageodo-ri, Sang Jae Suh Coll.; **Identification:** identifiedBy: Donguk Kim; **Event:** eventDate: 14.VIII.2023; **Record Level:** basisOfRecord: Dried Specimens

##### Notes

This species was collected during visual searches of wooden fences along the hiking trails in mid-mountainous areas (Fig. 2C, Fig. 3F and Fig. 7B). It was first recorded in Gageodo Island by Hwang (2015).

#### Chrysomelidae

93FBBA36-A29C-5291-8B8F-F081EACA5F52

#### Chrysomelinae

3D248D14-A1E7-548D-9B78-85DBE4F2A3F5

#### Doryphorini

F1AEF867-E00F-5EBD-9683-345089D59F8B

#### Chrysolina (Anopachys) aurichalcea

(Mannerheim, 1825)

C7AA7FCB-8710-5923-9A43-57CF1195D0E3

##### Notes

Shin and Noh (1970) .

#### Criocerinae

9022CD1F-FD86-5D32-9934-01DC7101057A

#### Lemini

6E738EA5-4DEF-566C-A605-1DF850E2DF1A

#### Lema sp.

1583B14E-3B1E-5C0F-ACAB-B36574EACE70

##### Notes

Shin and Noh (1970) .

#### Galerucinae

21A1A66E-8621-5A64-8C3F-C7E33237D44E

#### Luperini

EF9606F5-82F0-5797-A90F-AAD272C62514

#### Charaea flaviventris

(Motschulsky, 1860)

B8BC65C4-865F-5CD4-9F88-CFAC173E091B

##### Notes

Shin and Noh (1970) .

#### Charaea sp.

207B8FB6-1C8C-5CC0-A9D9-801AA3A1A256

##### Notes

Shin and Noh (1970) .

#### Coccinellidae

7E2D0861-B211-582D-85C0-5941CEC55A6F

#### Coccinellinae

CC5D8331-0A08-52E9-9E3D-C34BEF93A1AF

#### Coccinellini

09C0BD09-A15D-51CB-91E1-413D1D6D192D

#### Harmonia axyridis

(Pallas, 1773)

F0204AA8-8E8D-537F-BE14-C97274AB67AD

##### Notes

Shin and Noh (1970) .

#### Epilachnini

1FB21D1B-BBFF-5358-85F9-185CD5FC7C38

#### Henosepilachna vigintioctomaculata

(Motschulsky, 1857)

B1EA52E0-658A-542E-862A-2692CBFD8FA6

##### Notes

Shin and Noh (1970) .

#### Platynaspidini

2CA88060-BB4C-5315-A14E-50B2B17CDCD4

#### Phymatosternus sp.

66DD3374-736A-55D6-88F7-680BEDA3AFD7

##### Notes

Shin and Noh (1970) .

#### Curculionidae

DB890039-0610-50E5-A754-F71326F6F3A9

#### Entiminae

0A3E2377-757D-53B7-B2AE-62153388AE23

#### Cyphicerini

213BBA4E-60F2-5F7D-9A19-BAA724CD3BD5

#### Calomycterus setarius

Roelofs, 1873

52173220-C157-5A77-8648-CC44731F02BE

##### Notes

Han et al. (2014) .

#### Sitonini

563AB08F-A2B3-5B9D-827C-DFBFDBCD9ED4

#### Eugnathus distinctus

Roelofs, 1873

6CAB14D5-68D4-547A-AD77-44585CB2933C

##### Notes

Shin and Noh (1970) .

#### Trachyphloeini

2005433A-02CC-5F43-B6A4-CBCFA8831B2C

#### Pseudocneorhinus bifasciatus

Roelofs, 1879

B9B8DD3D-F174-55EB-9366-463832BB98AE

##### Notes

Shin and Noh (1970) .

#### Pseudocneorhinus soheuksandoensis

Han & Yoon, 2000

5682BB94-EFD3-5E01-BF5A-2A08154BAFE7

##### Notes

Han and Yoon (2000), Honam National Institute of Biological Resources (HNIBR) (2022).

#### Lixinae

7B033991-2C8B-5331-8A8F-B7857BF1300E

#### Lixini

556BD19F-6BAE-5A81-9C94-4A5EA21DA191

#### Lixus (Dilixellus) depressipennis

Roelofs, 1873

2C8EB166-7B5E-5F32-A418-327CCFB1B846

##### Notes

Shin and Noh (1970) .

#### Lixus (Dilixellus) maculatus

Roelofs, 1873

B33A5115-3113-59A9-911A-E15C88D6495F

##### Notes

Honam National Institute of Biological Resources (HNIBR) (2022) .

#### Molytinae

ECB51B71-5C70-58CB-8963-805748FABF5C

#### Hylobiini

E8433D70-5121-543D-A0E1-6A0020961ACA

#### Pimelocerus perforatus

(Roelofs, 1873)

F6760521-8FE0-5AC4-A246-BFD578C81649

##### Notes

Shin and Noh (1970) .

#### Scolytinae

67A0E84C-3B27-58EC-ABB3-84C919A60F55

#### Scolytini

61BAAACD-3355-5E01-94FF-EB48D00DE5B4

#### Scolytus frontalis

Blandford, 1894

710AF75B-E425-5B0F-9026-E2A54457FF98

##### Notes

Shin and Noh (1970) .

#### Scolytus esuriens

Blandford, 1894

6493043E-8B03-5443-9000-2D71FF39CD54

##### Notes

Shin and Noh (1970) .

#### Elateridae

4727C597-BABC-5E52-BA9A-CA24385E5FAC

#### Agrypninae

B97EA065-FF09-5AE9-8937-519390A7C2A2

#### Agrypnini

E8A065C0-6D75-5FFD-A0BA-3C91EBC9C183

#### Agrypnus binodulus coreanus

Kishii, 1961

281B443A-91A2-5093-ACDB-B77854D90473

##### Notes

Honam National Institute of Biological Resources (HNIBR) (2022) .

#### Elaterinae

DA4214D2-B06A-5780-BA97-914D824D3A47

#### Melanotini

0CED91FE-CF7A-50E5-9496-C9DA5B97493D

#### Melanotus (Melanotus) legatus legatus ​​​​​​​

Candèze, 1860

17D9B134-1CB9-581B-A86F-DCE20FD4FDF4

##### Notes

Shin and Noh (1970), Honam National Institute of Biological Resources (HNIBR) (2022).

#### Lucanidae

1C189A4A-FDFB-5FD2-8D6F-C1BBEC733244

#### Lucaninae

51C10E9A-9FA1-568A-84BE-E8EC30863B22

#### Aegini

5B1A1D8E-F9CA-5241-9ABD-31199D51FBBA

#### Aegus laevicollis subnitidus

Waterhouse, 1873

EE4AF4D2-6D7F-56D1-BF0D-5F9DE71CAD1D

##### Notes

Shin and Noh (1970), Kim and Kim (2010), Kim and Kim (2014), Honam National Institute of Biological Resources (HNIBR) (2022).

#### Dorcini

BEEC633B-5C4E-571B-8E4F-412216FB6DAE

#### Dorcus consentaneus consentaneus

(Albers, 1886)

C994BEA1-513E-5E4F-9A36-F9F84768CEA4

##### Materials

- **Type status:** Other material. **Occurrence:** sex: 1 male; occurrenceID: ACCCBB97-7CEB-5B50-AB06-AE5D0BA55B68; **Location:** island: Gageodo Island; country: South Korea; stateProvince: Jeollanam-do; locality: Shinan-gun, Heuksan-myeon, Gageodo-ri, Donguk Kim leg.; **Identification:** identifiedBy: Donguk Kim; **Event:** eventDate: 28.VII.2015; **Record Level:** basisOfRecord: Dried Specimen
- **Type status:** Other material. **Occurrence:** sex: 11 males, 9 females; occurrenceID: ACCCBB97-7CEB-5B50-AB06-AE5D0BA55B68; **Location:** island: Gageodo Island; country: South Korea; stateProvince: Jeollanam-do; locality: Shinan-gun, Heuksan-myeon, Gageodo-ri, Sang Jae Suh Coll.; **Identification:** identifiedBy: Donguk Kim; **Event:** eventDate: 13.VIII.2023; **Record Level:** basisOfRecord: Dried & Ethanol-Preserved Specimens

##### Notes

**Figure 8.**
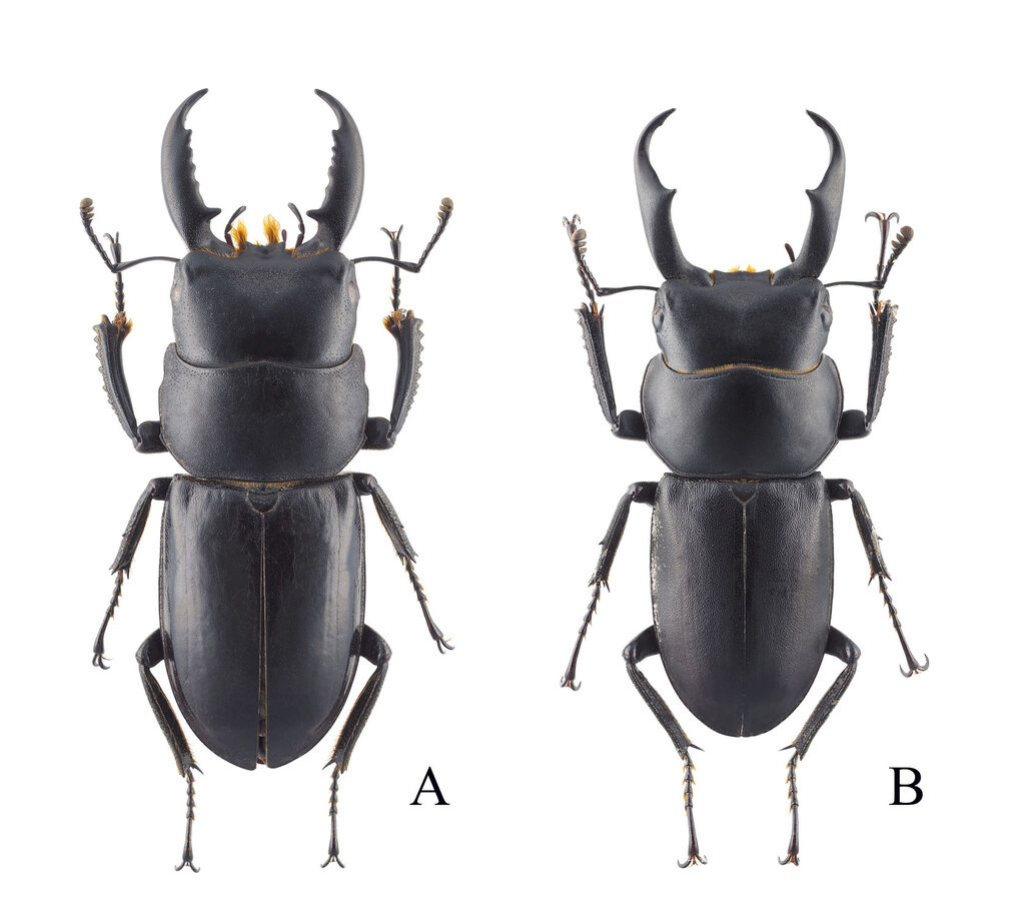
Lucanid species from Gageodo Island. **A** *Dorcusconsentaneusconsentaneus*; **B** *Dorcusrectusrectus*.

Most of the individuals (n = 16) were collected in pit-fall traps in the mid-mountainous areas of Mt. Doksilsan. Some individuals (n = 4) were observed feeding on the sap of *Machilusthunbergiai* (Lauraceae) growing on mountain ridges or walking on forest roads (Fig. 3G and Fig. 8A). This is the first record of this species on Gageodo Island.

#### Dorcus rectus rectus

(Motschulsky, [1858])

1CE234A8-9FA6-5DF9-AC35-6EA278936937

##### Materials

- **Type status:** Other material. **Occurrence:** sex: 1 male, 1 female; occurrenceID: 93375A2C-CD19-534E-9F32-FE324667D607; **Location:** island: Gageodo Island; country: South Korea; stateProvince: Jeollanam-do; locality: Shinan-gun, Heuksan-myeon, Gageodo-ri, Donguk Kim leg.; **Identification:** identifiedBy: Donguk Kim; **Event:** eventDate: 28.VII.2015; **Record Level:** basisOfRecord: Dried Specimens
- **Type status:** Other material. **Occurrence:** sex: 8 males, 10 females; occurrenceID: 276ED401-6C1B-5AD3-8DDA-FD8301D65DAE; **Location:** island: Gageodo Island; country: South Korea; stateProvince: Jeollanam-do; locality: Shinan-gun, Heuksan-myeon, Gageodo-ri, Sang Jae Suh Coll.; **Identification:** identifiedBy: Donguk Kim; **Event:** eventDate: 26.V.2023; **Record Level:** basisOfRecord: Dried & Ethanol-Preserved Specimens

##### Notes

This species was collected during visual searches and in pit-fall traps. It was widely observed on forest roads from the lowland to the highland areas of Gageodo Island and some individuals (n = 8) were also attracted to street lights in low-lying coastal areas (Fig. 3H, I and Fig. 8B). This is the first record of this species on Gageodo Island.

#### Figulini

7B685AFB-2BEE-5576-83EE-980D3BA37964

#### Figulus binodulus

Waterhouse, 1873

6A439CE0-9D3A-53DA-9DD3-94E5D33D9E07

##### Notes

Kim et al. (2019) .

#### Figulus punctatus punctatus

Waterhouse, 1873

43CD2287-A3C3-5963-848E-9BB6087C8BA3

##### Notes

Kim and Kim (2014), Kim et al. (2019).

#### Melyridae

C48A242D-8AAC-5224-81D9-BB8403DC36C3

#### Malachiinae

A0FD8197-C5C4-5CAF-BDF1-CAB9A5047BDD

#### Malachiini

895B80EC-AFDF-5C21-A29E-AE8C076E1A64

#### Intybia tsushimensis

(Satô & Ohbayashi, 1968)

5BAC8D3D-95AE-5B62-8133-115651E9EBC4

##### Materials

- **Type status:** Other material. **Occurrence:** sex: 2 males, 1 female; occurrenceID: 1C14E266-6849-5024-B9F1-5DFEE7B5C847; **Location:** island: Gageodo Island; country: South Korea; stateProvince: Jeollanam-do; locality: Shinan-gun, Heuksan-myeon, Gageodo-ri, Sang Jae Suh Coll.; **Identification:** identifiedBy: Donguk Kim; **Event:** eventDate: 13.VIII.2023; **Record Level:** basisOfRecord: Dried Specimens

##### Notes

This species was first recorded in Korea by Kang (2014) and subsequently listed in the national Korean insect checklist (National Institute of Biological Resources (NIBR) 2019, Korean Society of Applied Entomology (KSAE) and The Entomological Society of Korea (ESK) 2021). However, it was determined to be a misidentification of *Intybiakishii* (Nakane, 1955), based on a reference image and diagnosis. Therefore, *I.tsushimensis* (Satô & Ohbayashi, 1968) is a new record for Korea, as revealed by this study. Our recent extensive survey has uncovered that *I.tsushimensis*, previously known as an endemic species of Japan (specifically Tsushima Island), is distributed throughout the Korean Peninsula (Kim and Suh, unpublished data). It was collected by sweeping along the evergreen broad-leaved forest roads in the mid-mountainous areas of Mt. Doksilsan (Fig. 2C).

##### Diagnosis

**Figure 9.**
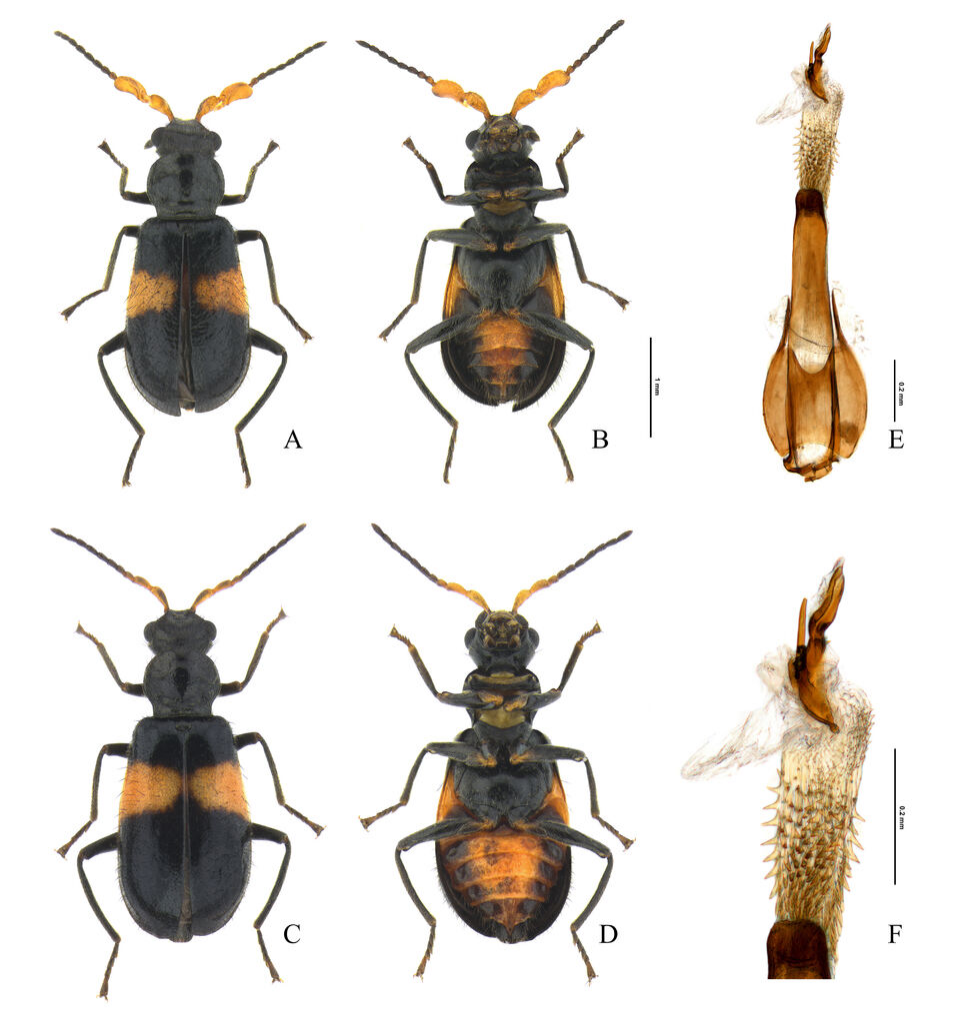
Melyrid species from Gageodo Island, *Intybiatsushimensis*. **A** male, dorsal view; **B** male, ventral view; **C** female, dorsal view; **D** female, ventral view; **E** male genitalia; **F** endophallus.

Elytra with a reddish-orange or yellowish fascia, slightly or gradually narrowed to suture and sometimes divided into two fasciae by suture; legs entirely black; antennae yellow, with black or dark brown antennomeres V–XI; endophallus without spinous plate; spinous area composed with short triangular spines (Fig. 9F).

#### Salpingidae

52774678-5A84-541F-96DF-425FA2F22586

#### Othniinae

01984258-8173-5A77-B8DD-66564A6D1488

#### Elacatis ocularis

(Lewis, 1891)

FF839BD2-24E0-55E5-8A88-C2BDC3C49094

##### Notes

Honam National Institute of Biological Resources (HNIBR) (2022) .

#### Scarabaeidae

DCC69A5E-7136-59E8-AFFF-25217B21D1C9

#### Aphodiinae

6B3AD4C5-1425-5718-848D-ABCF3FED0D46

#### Aphodiini

FC8AAEEC-2D3B-507B-A750-6D56677378E3

#### Aphodius (Aganocrossus) urostigma

Harold, 1862

E33EFC4F-D1DA-5550-9494-EE28DDBADB12

##### Notes

Kim (2012) .

#### Aphodius (Aphodius) elegans

Allibert, 1847

64E29664-D96B-5736-AE17-15F6F258B411

##### Notes

Kim (2012) .

#### Cetoniinae

CF38F270-9455-5E31-BA53-28B026D88F4A

#### Cetoniini

C05BA0DD-952D-5707-A43D-0BE1EAF218E0

#### Cetonia (Eucetonia) pilifera pilifera

(Motschulsky, 1860)

81070129-1C68-5A72-BF66-33106EB9DBD3

##### Notes

Honam National Institute of Biological Resources (HNIBR) (2022) .

#### Gametis jucunda

(Faldermann, 1935)

8461CA93-4C8F-5C1C-8507-B3F189FFCE32

##### Notes

Honam National Institute of Biological Resources (HNIBR) (2022) .

#### Protaetia brevitarsis seulensis

(Kolbe, 1886)

D035FED9-3FF8-562E-A6CF-8AF23788F5B5

##### Notes

Honam National Institute of Biological Resources (HNIBR) (2022) .

#### Protaetia orientalis submarmorea

(Burmeister, 1842)

1AF13B3E-8256-51E7-A844-3F9B8F56190B

##### Notes

Shin and Noh (1970), Kim (2011).

#### Dynastinae

C0E4797D-B752-5DB6-922C-B1DFDF91F578

#### Phileurini

6076CD00-1339-58E8-A23C-62B6B7C3DED4

#### Eophileurus chinensis

(Faldermann, 1835)

21A303D4-D2EF-5F94-B74A-CDCF62BDD381

##### Notes

Kim (2011) .

#### Melolonthinae

00B2A8DC-CF32-5732-8EFD-18CB84FDB981

#### Melolonthini

918FADF1-56F7-5C4D-B1D4-AA1736D947A8

#### Holotrichia diomphalia

(Bates, 1888)

233750E1-AA6C-5C1B-B480-1E4D5C7D7A37

##### Notes

Kim (2011) .

#### Sericini

4AA2D3AB-209B-58B4-A5DB-A389BF5ABC67

#### Maladera fusania

(Murayama, 1934)

E65A4D9D-B05D-553D-B20D-37814F6EB2D3

##### Notes

Kim (2011) .

#### Maladera holosericea

(Scopoli, 1772)

0A579806-8E31-5BE9-9C70-4A97BD46E487

##### Notes

Kim (2011) .

#### Ruetelinae

686BA558-945C-55CC-B3FF-BEDAE7FB4B8E

#### Anomalini

1D9F7162-8CA7-5B7F-96D9-A26000E4F341

#### Anomala albopilosa

Hope, 1839

5A119466-5E42-5D9B-BE31-2A9315A33B0E

##### Notes

Shin and Noh (1970), Kim (2011).

#### Mimela splendens

(Gyllenhal, 1817)

13E5D813-F2AB-537B-9714-B5DEDD59250A

##### Notes

Honam National Institute of Biological Resources (HNIBR) (2022) .

#### Scarabaeinae

C9FF772A-4559-5848-99AB-FB598556EE03

#### Onthophagini

4CE63906-3B22-594C-9B7E-E31FA5BEC18D

#### Onthophagus (Strandius) lenzii

Harold, 1874

DD34B018-E26C-5C4C-8D30-B506D3206A30

##### Notes

Shin and Noh (1970) .

#### Staphylinidae

968999C7-439D-5EA7-BD33-12B2167B19AB

#### Pselaphinae

3A2A65A3-6C88-5281-B4F1-CC446580D3A1

#### Bythinini

AC028D19-8E59-5388-B4CE-D49F5704F7B4

#### Bryaxis nemorosus

Choi, Park, Lee & Park, 2023

6487AE26-6543-53C4-B37F-58CDD09C1A2F

##### Notes

Choi et al. (2023) .

#### Silphinae

F205F092-24B1-53A7-816E-421102F28D19

#### Necrophila (Eusilpha) jakowlewi jakowlewi

(Semenov, 1891)

E35546AD-FEB9-5362-A36B-67599210EB00

##### Notes

Cho (2013), Choi et al. (2018).

#### Necrodes littoralis

(Linneaus, 1758)

0952D10F-EF31-55D6-A09B-426AF85F800D

##### Notes

Cho (2013), Choi et al. (2018).

#### Silpha (Silpha) perforata

Gebler, 1832

1E155722-70CD-5FC6-BB70-C5624D050FCC

##### Notes

Shin and Noh (1970), Honam National Institute of Biological Resources (HNIBR) (2022).

#### Staphylininae

E1AA2F7C-99DC-5659-B52F-F30430769E67

#### Staphylinini

1FFD8FB1-0FF6-5F3C-BD5B-1D445308EA37

#### Ocypus (Pseudocypus) lewisius

Sharp, 1874

F81FD549-1A3B-5122-A545-2333383B1CE2

##### Notes

Cho (2019) .

#### Philonthus (Philonthus) sublucanus

Herman, 2001

EC3BC060-9B71-56C8-A73E-AE44F949D25D

##### Notes

Cho (2014) .

#### Platydracus (Platydracus) plebejus

(Bernhauer, 1915)

851BD279-0027-5172-87B6-E42CF010E449

##### Notes

Cho (2019) .

#### Platydracus sp.

40B7FB57-4C8B-570C-A434-524EA8845A15

##### Notes

Shin and Noh (1970) .

#### Tenebrionidae

C42A0DA4-47F5-5B8F-A709-C84A1DB35238

#### Tenebrioninae

4B1BFF55-1262-5B81-BA1B-B0BAB639583F

#### Opatrini

035A8361-AFBC-54F8-97CC-CB48772ACFC4

#### Gonocephalum (Gonocephalum) coenosum

Kaszab, 1952

C4FC81C8-7C67-54D1-8776-74B0A155A39D

##### Notes

Shin and Noh (1970), Kim and Kim (2000), Jung (2012), Honam National Institute of Biological Resources (HNIBR) (2022).

#### Pedinini

1C77C829-DD9C-55D8-AC51-8B07196CDD24

#### Blindus strigosus

(Faldermann, 1835)

EFF60C4B-031F-523C-A391-818494F08E13

##### Notes

Jung (2012) .

#### Ulomini

9EC03F28-B8E5-56B4-88D1-6D44D235EB84

#### Uloma sp.

58614C62-C977-51D4-A3E9-52AD86057DC8

##### Notes

Shin and Noh (1970) .

## Analysis

### Composition of Coleoptera on Gageodo Island

**Figure 10.**
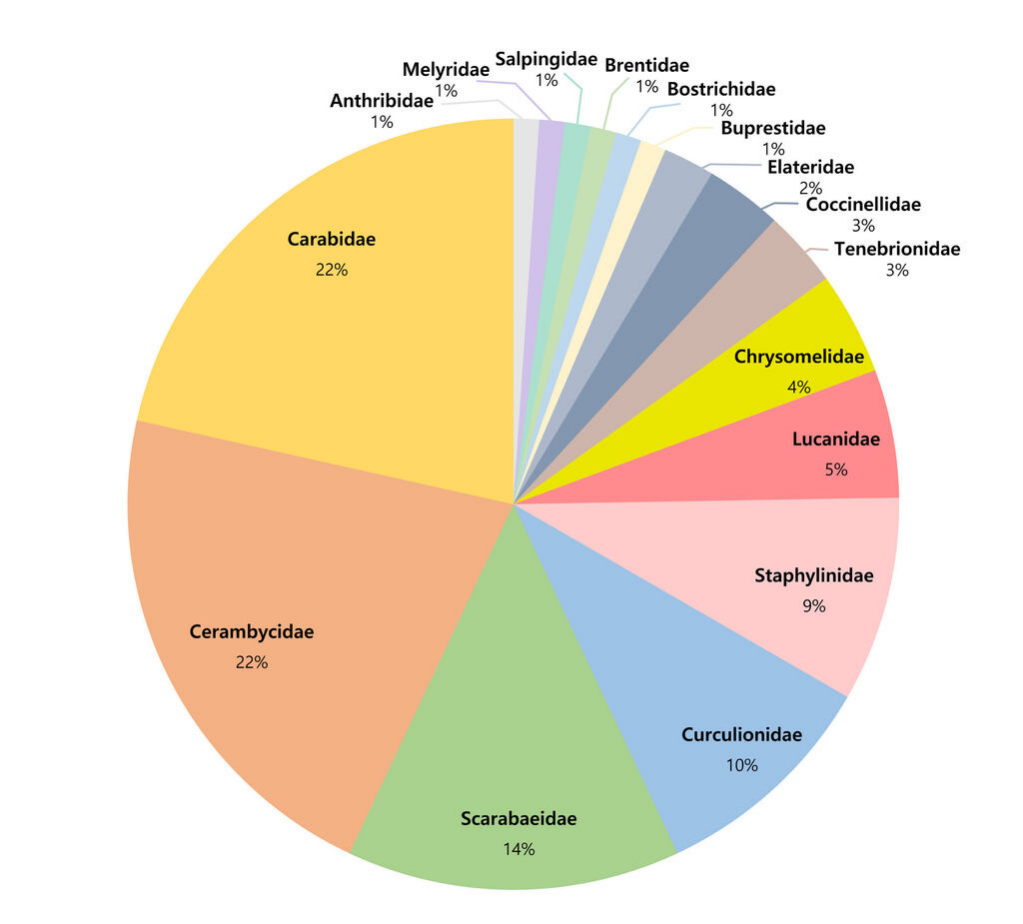
Composition of the coleopteran families on Gageodo Island.

In this study, the presence of Buprestidae, Carabidae and Melyridae on Gageodo Island was confirmed for the first time and 31 species from the five investigated coleopteran families were newly added to the fauna of Gageodo Island. Furthermore, after combining the results from previous studies that have used samples from Gageodo Island, a list of 93 species within 16 families was constructed. Of these families, Carabidae and Cerambycidae demonstrated the highest species diversity, with 20 species each (22%), followed by Scarabaeidae with 13 species (14%), Curculionidae with nine species (10%) and Staphylinidae with eight species (9%) (Fig. 10).

## Discussion

### Notes on the coleopterans on Gageodo Island

Buprestid and cerambycid beetles are involved in highly specialised interactions with host plants and the species composition and diversity of these taxa are affected by plant diversity (Evans et al. 2007, Fahri et al. 2016, Pérez-Hernández et al. 2022). Gageodo Island has unique floristic characteristics (Yang et al. 2013, So et al. 2014, Oh et al. 2015); thus the diversity of phytophagous beetles may also be high on the Island. However, only one species of buprestid beetle was found during our surveys and it is the only species recorded to date on the Island. The weather conditions during the surveys, including overcast skies and rain, may have affected the results since adult buprestids are diurnal and photophilic (Evans et al. 2007). The Island has various host plants for buprestid beetles, including *Catanopisissieboldii* (Fagaceae), *Quercusacuta* (Fagaceae), *Machilusthunbergia* (Lauraceae) and *Rubusbuergeri* (Rosaceae), indicating the potential for the discovery of more species from genera such as *Agrilus*, *Trachys*, *Habroloma* etc. (Yang et al. 2013, Ohmomo and Fukutomi 2013).

**Figure 11.**
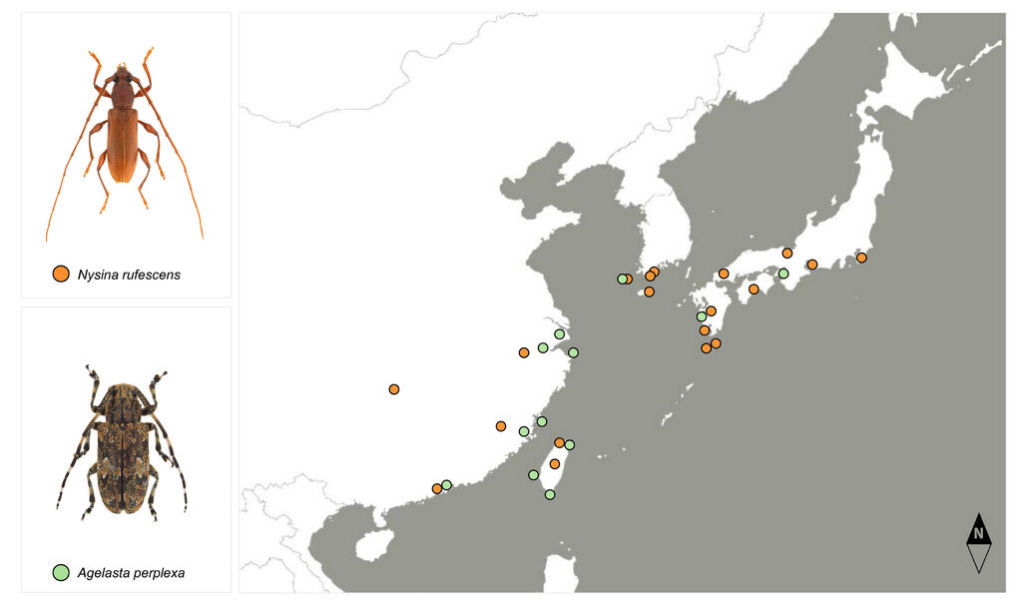
Distribution map of *Agelastaperplexa* (green dots) and *Nysinarufescens* (orange dots). The map was taken from Snazzy Maps (https://snazzymaps.com) and edited using Adobe Photoshop 21.2.0 (Adobe Systems Inc.). (Sources: Hayashi et al. (1984), Jang et al. (2015), Lee and Lee (2021); GBIF [http://www.gbif.org]; INaturalist [https://www.inaturalist.org]).

Cerambycid beetles have been well-studied on the Korean Peninsula (e.g. Lee (1987), Jang et al. (2015), Hwang (2015) and Lee and Lee (2021)), with over 350 species within six subfamilies (Prioninae, Lepturinae, Spondylidinae, Necydalinae, Cerambycinae, Lamiinae) recognised to date. Amongst the species found on Gageodo Island, *Agelastaperplexa* and *Nysinarufescens* are only known from the insular region of the Korean Peninsula. In particular, in Korea, *A.perplexa* exhibits a unique distribution limited to Gageodo Island and the northernmost distribution limit zone for both species extends to South Korea and Japan in East Asia (Fig. 11).

Carabid beetles represent a species-rich taxon, with over 700 species and subspecies documented on the Korean Peninsula (Korean Society of Applied Entomology (KSAE) and The Entomological Society of Korea (ESK) 2021). Though faunistic surveys of this family have been frequently conducted, they have been restricted to mountainous regions (Jung et al. 2012, Jung et al. 2013, Lee et al. 2014, Jung et al. 2015, Hong et al. 2017) and Jejudo Island (Paik 1988, Paik and Kwon 1993, Paik 1997, Paik and Jung 2003, Paik and Jung 2004, Honam National Institute of Biological Resources (HNIBR) 2022); thus surveys on other islands need to be conducted. The present study found 20 carabid species new to the fauna of Gageodo Island. Of these, *Chlaeniuskurosawai* is new to the insular region of the Korean Peninsula (Honam National Institute of Biological Resources (HNIBR) 2022). This record makes Gageodo Island the westernmost record for its distribution range. As carabid beetles occupy diverse habitats and microhabitats (Arndt 2005, Kotze et al. 2011), further surveys with a more diverse range of collection methods (e.g. light and malaise traps) may lead to a better understanding of the carabid fauna of the Island.

Lucanid beetles are another well-studied group in Korea, with a total of 16 species recorded on the Korean Peninsula (Kim and Kim 2014, Kim et al. 2019). Of these, *Dorcusrectusrectus* and *Dorcustitanuscastanicolor* are the most common species and are distributed across the Peninsula, including insular regions (Kim and Kim 2014, Kim et al. 2019, Honam National Institute of Biological Resources (HNIBR) 2022). In particular, *D.titanuscastanicolor* is found on islands such as Baekryeongdo, Jejudo and Ulleungdo Island, which are distant from the mainland (Kim and Kim 2014). It is also known to inhabit islands near Gageodo Island, such as Hongdo (Kim and Kim 2014) and Jindo (Kim and Suh, unpublished data). However, mysteriously, it has not yet been confirmed to be present on Gageodo Island (Kim and Kim 2014, Kim et al. 2019, Honam National Institute of Biological Resources (HNIBR) 2022). *Dorcusconsentaneus*, which has a similar life history to *D.titanuscastanicolor* (Kim and Kim 2014) was typically dominant on the Island along with *D.rectus*.

Melyrid beetles are generally a common, but poorly studied group in Korea, with only a few studies having been conducted (Kang 2014, Ikeda and Yoshitomi 2017, Lee et al. 2020). In the present study, *Intybiatsushimensis*, previously known as an endemic species of Japan (specifically Tsushima Island), was confirmed to be present in Korea for the first time, following a mistaken record, representing the south-westernmost occurrence of the species.

Other species-rich coleopteran families such as Curculionidae (weevils), Chrysomelidae (leaf beetles) and Staphylinidae (rove beetles) remain underinvestigated and thus require further surveys.

### Species of interest

Climate change has a significant impact on the geographical distribution of biological organisms globally and selecting indicator species can be used to make informed and effective ecosystem management decisions (Kelly and Goulden 2008, Lee et al. 2010, Siddig et al. 2016, Liang et al. 2018). Accordingly, 100 climate-sensitive biological indicator species (CBIS), encompassing vertebrates, invertebrates and plants, were designated to monitor the influence of climate change on the distribution of biological species on the Korean Peninsula (Lee et al. 2010). Of the beetle species identified on Gageodo Island, two cerambycid species, *Agelastaperplexa* and *Nysinarufescens*, could be considered CBIS candidates, based on the criteria proposed by Lee et al. (2010). The northernmost distribution limit zone for these species is South Korea and Japan in East Asia (Fig. 11); thus their distribution limits in this area hold important biogeographical value. Moreover, their host plants are associated with the warm temperate evergreen broad-leaved tree species *Machilusthunbergii* (Lauraceae), which has been reported to be extending its northern distribution limit range (Shin et al. 2022). Considering their distribution and host plants, there is a possibility that their habitat boundaries may expand due to climate change on the Korean Peninsula. Continuous monitoring efforts are required to assess their possibility as a climate indicator and to track habitat boundary shifts in response to climate change.

### Conclusion

The Korean Peninsula has great potential as a study area for furthering the understanding of island biogeography due to its numerous islands of various sizes and varying distances from the mainland. Gageodo Island is the south-westernmost island, thus its floristic and faunistic data are particularly valuable for understanding Korean island biogeography. This study discovered previously unrecorded coleopteran species on the island in Korea and established the first organised Coleoptera checklist, providing foundational insights into the coleopteran fauna of this biogeographically important island and serving as a basic resource for future research.

## Figures and Tables

**Table 1.** Historical review of the reports of coleopteran species from Gageodo Island (species first described from the Island are marked with an asterisk).

**Reference**	**Species**
Shin and Noh (1970)	Chrysomelidae: *Exosomaflaviventris* (=*Charaeaflaviventris*), *Exosoma* sp. (=*Charaea* sp.), *Chrysolinaaurichalca* (*sic.*=*C.aurichalcea*), *Lema* sp.; Coccinellidae: *Harmioniaaxyridis* (*sic.*=*Harmoniaaxyridis*), *Epilachnaigintioctomaculata* (=*Henosepilachnaigintioctomaculata*), *Platynasis* sp. (=*Phymatosternus* sp.); Curculionidae: *Callirhopalusbifasciatus* (=*Pseudocneorhinusbifasciatus*), *Callirhopalusobesus* (=*Pseudocneorhinussoheuksandoensis*), *Eugnathusdistinctus*, *Hylobiuscribripennis* (=*Pimelocerusperforatus*), *Lixussipressipennis* (=*Lixusdepressipennis*), *Scolytusfrontalis*, *Scolytusesuriens*; Elateridae: *Melanotuslegatus*; Lucanidae: *Aeguslaevicollis*; Scarabaeidae: *Anomalaalbopilosa*, *Onthophaguslenzii*, *Potosiaaerata* (=*Protaetiaorientalissubmarmorea*); Staphylinidae: *Silphaperforata*, *Platydracus* sp.; Tenebrionidae: *Gonocephalumterminalae* (*nec.* =*Gonocephalumcoenosum*), *Ulma* sp. (*sic.* =*Uloma* sp.)
Lee (1987)	Cerambycidae: *Chlorophorusmuscosus*, *Pterolophiamultinotata* (=*Pterolophiaangustamultinotata*), *Ropicacoreana*
Kim and Kim (2000)	Tenebrionidae: *Gonocephalumcoenosum*
Han and Yoon (2000)	Curculionidae: Pseudocneorhinussoheuksandoensis*
Kang (2002)	Cerambycidae: *Mesosaperplexa* (=*Agelastaperplexa*)
Kim (2011)	Scarabaeidae: *Eophileuruschinensis*, *Holotrichiadiomphalia*, *Maladerafusania*, *Maladeraholosericea*
Jung (2012)	Tenebrionidae: *Blindusstrigosus*
Kim (2012)	Scarabaeidae: *Aphodiusurostigma*, *Aphodiuselegans*
Park et al. (2012)	Anthribidae: *Tropideresnaevulus*
Han et al. (2013)	Brentidae: *Sergiolagriseopubescens*
Cho (2014)	Staphylinidae: *Philonthussublucanus*
Kim and Kim (2014)	Lucanidae: *Figuluspunctatus*
Han et al. (2014)	Curculionidae: *Calomycterussetarius*
Jang et al. (2015)	Cerambycidae: *Nysinarufescens*, *Perissuskimi*, *Rondibilisundulata*
Hwang (2015)	Cerambycidae: *Exocentruslineatus*, *Lepturaannularisannularis*, *Psephactusremigerremiger*, *Pterolophiaannulata*
Choi et al. (2018)	Staphylinidae: *Necrophilajakowlewi*, *Necrodeslittoralis*
Cho (2019)	Staphylinidae: *Ocypuslewisius*, *Platydracusplebejus*
Kim et al. (2019)	Lucanidae: *Figulusbinodulus*
Honam National Institute of Biological Resources (HNIBR) (2022)	Cerambycidae: *Agapanthiaamurensis*, *Moechotypadiphysis*; Curculionidae: *Lixusmaculatus*; Elateridae: *Agrypnusbinoduluscoreanus*; Salpingidae: *Elacatisocularis*; Scarabaeidae: *Cetoniapiliferapilifera*, *Gametisjucunda*, *Mimelasplendens*, *Protaetiabrevitarsisseulensis*
Choi et al. (2023)	Staphylinidae: Bryaxisnemorosus*
Park et al. (2024)	Bostrichidae: *Melalgusbatillus*
